# Integrated Metabolomics and Selection Signal Analysis Provide Insights Into the Selection for Flavonol Biosynthesis Associated With Lettuce Quality Improvement

**DOI:** 10.1111/pbi.70716

**Published:** 2026-07-13

**Authors:** Xiao Yang, Shiwei Wei, Ge Jin, Li Zhang, Ran Du, Yajun Wang, Chengcheng Shen, Tao Huang, Guotao Huo, Jie Peng, Bo Song, Fangyuan Zhang, Victor Hugo Escalona Contreras, Lijun Luo, Yuejian Li, Francisco A. Tomas‐Barberan, Bin Liu, Qichang Yang

**Affiliations:** ^1^ Institute of Urban Agriculture, Chinese Academy of Agricultural Sciences, Chengdu National Agricultural Science and Technology Center Chengdu China; ^2^ Shanghai Agrobiological Gene Center Shanghai China; ^3^ Agricultural Genomics Institute at Shenzhen, Chinese Academy of Agricultural Sciences Shenzhen China; ^4^ School of Life Sciences Southwest University Chongqing China; ^5^ Biological Breeding Laboratory Xinjiang Uygur Autonomous Region Academy of Agricultural Sciences Urumchi China; ^6^ Faculty of Agricultural Sciences University of Chile Santiago Chile; ^7^ Quality, Safety, and Bioactivity of Plant Foods CEBAS‐CSIC Murcia Spain

**Keywords:** domestication, flavonol biosynthesis, *Lactuca sativa*, non‐targeted metabolomics, quality improvement, selection signal

## Abstract

Lettuce is a critical leafy vegetable consumed worldwide and is a substantial dietary source of health‐promoting compounds. Exploring changes in metabolism during lettuce domestication under artificial selection conditions is important to facilitate further breeding and cultivation for quality improvement. A liquid chromatography‐mass spectrometry‐based metabolomics approach was used to putatively identify 237 metabolites from 40 accessions, of which 130 were identified as metabolite identification level 1. Subsequently, 29 metabolites linked to lettuce quality improvement and their potential associated genes involved in lettuce domestication and differentiation were analysed. Muti‐omics approach showed that metabolites involved in flavonol biosynthesis are the main metabolic distinctions between wild and modern cultivars, which is attributable to the selection signal observed in *LsF3*′*H*, a key enzyme involved in the catalysis of flavonoid hydroxylation at the 3′‐position. These findings provide a comprehensive view of quality‐ and flavour‐related metabolite variation in lettuce, reveal the potential for quality improvement associated with flavonol biosynthesis, and offer valuable insights into the genetic basis for improving lettuce flavour and nutrition.

## 1 Introduction

Lettuce, the most widely consumed leafy vegetable worldwide, is commercially available throughout the year and can be cultivated in open fields, greenhouses or plant factories (Decardi‐Nelson and You 2024; Yuan et al. 2025). The annual lettuce (and chicory) production was approximately 27.0 million tons in 2021, with a gross production value of 16.5 billion US dollars according to the statistics of the United Nations Food and Agriculture Organization. Lettuce is a rich source of fibres, vitamins and health‐promoting compounds, including phenolic acids, flavonoid glycosides and sesquiterpene lactones (Yang et al. 2022). These health‐promoting compounds observed in lettuce have different health benefits, including preventive effects against certain cancer types and cardiovascular diseases (Nicolle et al. 2004; Jung et al. 2013; Parmenter et al. 2025). Due to minimal processing, lettuce retains its nutritional value and bioactivity and has become a popular ready‐to‐eat vegetable that is widely consumed in regional cuisines such as Chinese hot pots, hamburgers, tacos and Korean‐style barbecue, where nutritional value and bioactivity are retained. Also, the flavour of lettuce is a critical factor for breeders, growers and consumers (Seo et al. 2009). Inadequate flavour, such as bitterness, has the capacity to influence the purchasing decisions of consumers (Park and Lee 2006). Therefore, exploring the potential for improving the nutritional value and flavour of lettuce to meet the growing demand for health benefits is of interest.

A substantial effort has been made to understand the genomes and transcriptomes of lettuce accessions and their wild ancestors, shedding light on the domestication history of cultivated lettuce (Reyes‐Chin‐Wo et al. 2017; Zhang et al. 2017; Wei et al. 2021). Lettuce underwent a single domestication event, and ancient cultivated lettuce originated approximately 10 800 years BP in the Fertile Crescent (Zhang et al. 2017). Modern lettuce cultivars descend from ancient Egyptian varieties, with domestication dating back at least 4500 years (de Vries 1997). As a popular vegetable worldwide, horticultural lettuce types are primarily grouped based on morphological characteristics such as butterhead, crisphead, looseleaf, romaine, stem and oilseed (Zhang et al. 2017). In recent years, studies on metabolite variation between lettuce cultivars and their wild relatives have been conducted, as nutritional properties are key characteristics of lettuce breeding (Zhang et al. 2024). For example, Yang, Wei, et al. (2018) reported that a non‐targeted metabolomic analysis of 30 lettuce varieties revealed metabolic adaptations to phenolic compounds resulting from artificial selection. van Treuren et al. (2018) investigated metabolite variation using an untargeted metabolomics approach on 150 *Lactuca* accessions from a gene bank; the 150 *Lactuca* accessions comprised 74 lettuce cultivars (crisphead, butterhead, leaf, romaine, cos, stalk and oilseed types of lettuce) and 76 wild *Lactuca* relatives ( *L. serriola* , *L. saligna* , *L. virosa* and *L. georgica*). This study provided a comprehensive understanding of the genetic diversity of phytochemicals among the accessions, including various phenylpropanoids (e.g., chlorogenic and chicoric acids) and flavonoids (e.g., quercetin conjugates). Zhang et al. (2020) showed that galactinol, malic acid, quinic acid and threonic acid might be the targets of domestication and/or influenced by local adaptation. However, how differences in the composition and concentration of lettuce metabolites, especially secondary metabolites involved in metabolic adaptations, nutritional values and flavour compositions, contribute to the evolutionary process of lettuce under artificial selection, remains unclear.

In the present study, we collected 40 accessions of wild and modern cultivated lettuce to compare genomic and metabolic differences between different cultivated types of lettuce and their wild ancestors. Comprehensive integrated metabolomics and high‐throughput genomic analyses associated with lettuce quality improvement were conducted. Subsequently, selection signal analysis was performed to identify the key genes linked to selection for lettuce quality improvement. These findings provide a comprehensive view of the metabolite diversity, metabolic pathway variations and potential quality improvements associated with flavonol biosynthesis in lettuce.

## 2 Methods

### 2.1 Cultivation of Plants and Assessment of the Morphological Characteristics

The collection of lettuce accessions used in this study comprised a core subset of high‐quality lettuce breeding projects, and the genome re‐sequencing data of the accessions belonging to the core subset of lettuce was uploaded to the SRA database at NCBI with accession number PRJNA1127331. The present dataset comprises the genomic re‐sequencing data of 274 accessions, including the main horticultural types, such as butterhead, crisphead, looseleaf, oilseed, romaine and wild relative species of lettuce. These accessions exhibit a high degree of genetic diversity and are distributed across a broad geographic range.

In this study, forty representative accessions were selected from the core subset of lettuce and subjected to metabolic profiling and genomic analysis. These accessions were 14 wild relatives, including nine *L. serriola* , one *L. virosa* and four intermediates. It also had 26 cultivars, including four butterheads, four crispheads, four loose leaves, three oilseeds and five romaine cultivars (Figure S1A; Dataset S1). The 40 selected accessions were broadly distributed across various phylogenetic branches and spanned the major genetic backgrounds of the entire population of 274 accessions, which indicated their representativeness and suitability for downstream analyses (Figure S1B).

Seeds of all 40 accessions were sown and germinated in the seedling trays with the nursing substrate (a 9:3:1 volume ratio mixture of turf, vermiculite and perlite). After 2 weeks, lettuce seedlings were transplanted and cultivated in a greenhouse at the Shanghai Agrobiological Gene Center in China (latitude 30°89′N, longitude 121°39′E) during the 2021 autumn season. The temperature was maintained at 22°C ± 3°C during the day and 15°C ± 2°C at night and the irradiance was 250–280 μmol·m^−2^·s^−1^ during the 14‐h photoperiod. Eight weeks later, lettuce plants were harvested and all leaves from six individual plants were collected and pooled as one biological replicate, and immediately flash‐frozen in liquid nitrogen, and stored at −80°C until further analysis. The morphological characteristics of the lettuce accessions used in this study were assessed in spring of 2020 following the morphological descriptors and evaluation criteria described by Li and Wang (2007).

### 2.2 Non‐Targeted Metabolomic Analysis

#### 2.2.1 Sample Preparation

Lettuce samples were extracted for liquid chromatography‐mass spectrometry (LC–MS) analysis as previously described with a slight modification (Yang, Wei, et al. 2018). Briefly, three independent biological replicates of lettuce leaf tissue (six plants per replicate) were lyophilized and ground into a fine powder using a freezer mill (JXFSTPRP, Jingxin, Shanghai, China). Lettuce powder (200 mg) was weighed and extracted with 1 mL of a solution of methanol and water (80:20, v/v) or 1 mL of an acidulated solution of methanol and water (80:19.5:0.5 v/v/v mixture of methanol, water and 0.1 M HCl) for negative or positive ion mode analysis, respectively. The samples were sonicated at 25°C for 30 min. Subsequently, samples were transferred to a refrigerator and kept at 4°C for 12 h, centrifuged at 12 000*g* for 10 min, and 0.5 mL of the supernatant was utilized for further analysis.

#### 2.2.2 LC–MS/MS Analysis

A Q Exactive Orbitrap mass spectrometer coupled with an Acquity Class ultra‐performance liquid chromatograph (UHPLC‐QE Orbitrap MS, Thermo Fisher Scientific, Waltham, MA, USA) with an HESI probe was used to analyse the metabolomes of lettuce samples in positive and negative modes (Fang et al. 2022). The analytical conditions were: injection volume, 1 μL; column, Kinetex Biphenyl column (150 mm × 2.1 mm, i.d.: 2.6 μm; Phenomenex, CA, USA.); temperature, 45°C. The mobile phases, water with 0.1% formic acid (A) and acetonitrile with 0.1% formic acid (B), were set at 0.25 mL·min^−1^. The gradient elution in the positive mode was set as: 0–2 min, 98% A; 2–8 min, 98% A; 8–12 min, linear gradient from 98% to 75% A; 12–18 min, linear gradient from 75% to 1% A, 18–18.1 min, 1% to 98% A; 18.1–20 min, 98% A. Finally, the initial conditions were maintained for 5 min to equilibrate the column. For the negative mode, the gradient elution was 0–2 min, 98% A; 2–8 min, linear gradient from 98%–75% A; 8–12 min, linear gradient from 75% to 1% A; 12–18 min, 1% A, 18–18.1 min, 1%–98% A; 18.1–20 min, 98% A. Finally, the initial conditions were maintained for 5 min to equilibrate the column.

The MS conditions included discharge current 6 μA; capillary temperature 320°C; heater temperature 250°C; sheath gas flow rate 35 Arb; auxiliary gas flow rate 10 Arb; scan range 80–1200 *m/z*, with spectra acquired in positive‐ and negative‐ion modes with data‐dependent acquisition MS scanning mode. Full‐scan and fragment spectra were acquired at high resolutions of 70 000 and 17 500, respectively. Several standard chemical mixtures, including amino acids, organic acids, phenolic compounds and sesquiterpenes, were initially injected to assist in lettuce metabolite identification (Dataset S2). In addition, quality control (QC) samples were prepared by mixing all samples and subsequently injected at the beginning, middle and end of each batch to evaluate analytical performance.

#### 2.2.3 Data Pre‐Processing and Metabolite Identification

Raw data acquired from UHPLC‐QE Orbitrap MS are stored as .raw files. These files contain multi‐level nested metadata (including instrument parameters, scan type, polarity mode, automatic gain control settings, ion injection time and resolution settings), scan headers, mass spectrum data blocks, a chromatographic retention time axis, a total ion current trace, as well as MS/MS fragmentation spectra and event lists for data‐independent acquisition. The raw data were then exported from the instrument manufacturer's software platform and uploaded to Progenesis QI (Waters Corp., Milford, MA, USA) for metabolomics data preprocessing, which included binning, reference selection for alignment, grouping, alignment and peak picking, as previously described (Garcia et al. 2020). Retention time (RT), *m/z*, MS^2^ fragments and isotopic distribution information were compared with those of the injected chemical standards, online databases with MS^2^ tags (e.g., Metlin, HMDB and Lipidmap), an in‐house lettuce metabolite library (Yang, Wei, et al. 2018; Xiang et al. 2025), and published studies for identifying lettuce metabolites. For data comparison, the MS and MS^2^ tolerances were set to 3 and 10 mDa, respectively. The relative abundance of each metabolite was subsequently calculated using Progenesis QI according to the response of the peaks in each column.

#### 2.2.4 Data Analysis of Acquired Metabolomics Data

The processed data were directly uploaded to the online software MetaboAnalyst (https://www.metaboanalyst.ca/), and the data matrix was analysed using univariate (fold changes, *t*‐test, volcano plots) and multivariate analyses such as principal component analysis (PCA), orthogonal partial least squares discriminant analysis (OPLS‐DA), K‐means clustering analysis (Euclidean distances, Ward clustering algorithm), and random forest classification (RF, with 500 trees, five predictors). To select differential metabolites between the modern and wild lettuce groups, a Venn diagram was constructed to identify biomarkers shared in three independent analyses. These analyses included the following: (1) metabolites with a variable importance in projection (VIP) score > 1.0 in the predictive component, (2) metabolites with a fold change > 2 and a *p*‐adjusted value < 0.05 and (3) the top 40 metabolites with the highest mean decrease in accuracy values in the RF analysis.

### 2.3 DNA Sequencing and Selection Signal Analysis

#### 2.3.1 DNA Sequencing

Genomic DNA was extracted from the lettuce samples using the cetyltrimethylammonium bromide technique (Doyle 1987). Sequencing libraries with an insert size of 400 bp were constructed and sequenced using an Illumina NovaSeq 6000 platform (2 × 150 bp paired‐end reads) by Shanghai Personalbio Co. Ltd., following the manufacturer's instructions. All 40 accessions of lettuce samples were sequenced with 5× average depth.

#### 2.3.2 Variant Calling, Filtering and Annotation

The clean paired‐end reads were aligned to the lettuce reference genome (https://phytozome‐next.jgi.doe.gov/info/Lsativa_V8) using the MEM algorithm implemented in BWA (version 0.7.17) software after trimming low‐quality reads using Trimmomatic (version 0.39). Picard tools (https://broadinstitute.github.io/picard/, version 2.27.5) were used to remove duplicate PCRs. Generated files in Binary Alignment Map format were used for indel realignment and to call single‐nucleotide polymorphisms (SNPs) via the RealignerTargetCreator, IndelRealigner and HaplotypeCaller modules of the Genome Analysis Toolkit (GATK, version 3.8) with default parameters.

A hard filtering step was applied based on the GATK Best Practices recommendation (‐‐filter‐expression “QD < 2.0 || MQ < 40.0 || QUAL < 30.0 || FS > 60.0 || SOR > 3.0 || MQRankSum < −12.5 || ReadPosRankSum < −8.0”) to obtain high‐confidence SNPs. Genotypes were subsequently phased and imputed using Beagle (version 4.1) based on genotype likelihood. Finally, the SNPs' functional effects were annotated using SnpEff (version 4.3).

#### 2.3.3 Investigating the Genetic Structure of the Population

The SNP dataset was further thinned via the following steps using VCFtools (version 0.1.16) and PLINK (version 1.9): (1) core chromosomes (“‐‐chr 1–9”); (2) minor allele frequency > 0.05 (‐‐maf 0.05); (3) missing values < 10% (‐‐max‐missing 0.9); (4) a Hardy–Weinberg equilibrium test (“‐‐hwe 0.001”); and (5) pruning SNPs by “‐‐indep‐pairwise 50 10 0.1”. Subsequently, 319 819 independent SNPs were used for further analysis. FastTree (version 2.1.11) was used to construct the maximum likelihood (ML) phylogenetic tree. PCA was conducted using PLINK (version 1.9). ADMIXTURE (version 1.3.0) was used to estimate individual ancestry by setting the number of ancestral clusters (K‐values) from 2 to 7. Nucleotide diversity (*π*) for each population was estimated using VCFtools (version 0.1.16) with a 50 kb window size and 5 kb sliding steps. Additionally, linkage disequilibrium (LD) decay with physical distance was calculated and visualized using PopLDdecay (version 3.43). Subsequently, TreeMix (version 1.1232) was utilized to model the genetic drift of genome‐wide allele frequency data to infer population splitting and mixing. Migration edges (with the “‐m” option) ranging from 0 to 5 were gradually added, and the standard errors of migration proportions were calculated using the “‐se” option.

#### 2.3.4 Identifying the Selective Sweeps

We used two complementary approaches to identify the genomic regions under selection. First, allele frequency differentiation between the wild and modern groups was estimated using BayeScan (version 2.0, with default parameters), which decomposed locus‐population F_ST_ coefficients into a population‐specific component (*β* value) and a locus‐specific component (α value) using a logistic regression model. A positive α value suggests diversifying selection, whereas a negative value implies balancing or purifying selection. Multiple testing correction in BayeScan was performed using a Bayesian framework that converts posterior probabilities into q‐values, thereby controlling the false discovery rate (FDR) value (Storey and Tibshirani 2003). SNPs with *q*‐value < 0.05 were considered as candidates for selection. Second, SweeD (version 4.0.0) was used to calculate the composite likelihood ratio (CLR) based on deviations from the neutral site frequency spectrum to detect selective sweeps within populations. The top 1% threshold was set to identify the significant sweep signals. Thus, regions simultaneously identified by BayeScan (high F_ST_) and SweeD (high CLR) were identified as demonstrating selection signatures. Furthermore, genes situated within these regions were functionally annotated using Gene Ontology terms and further analysed for pathway enrichment using the Kyoto Encyclopedia of Genes (KEGG) and Genomes (GO) database.

### 2.4 Gene Functional Study

The promoter sequences of the candidate gene in all accessions were extracted by using bcftools v1.17. A phylogenetic tree of promoters was constructed by FastTree v2.1.11. Multiple sequence alignment was performed using DNAMAN software (Lynnon Biosoft, Quebec, Canada) with the ClustalW algorithm under default parameters. *Cis*‐acting regulatory element analysis of the promoter sequences was performed using the PlantCARE database (https://bioinformatics.psb.ugent.be/webtools/plantcare/html/). Allelic knock‐out mutants of *Lsat_1_v5_gn_5_23101* (*LsF3*′*H*) were constructed via CRISPR/Cas9 gene editing using the looseleaf lettuce cultivar ‘jingyan yidali’ lettuce by Jiangsu Weimi Biotechnology Co. Ltd. The procedure was as follows. For CRISPR/Cas9‐mediated mutagenesis, two sgRNAs targeting the *LsF3′H* gene were designed using the CRISPR‐P online tool (http://crispr.hzau.edu.cn/CRISPR2/) and cloned into a CRISPR/Cas9 binary vector using a homologous recombination‐based cloning method. This vector carries the *aadA* (aminoglycoside adenyltransferase) gene as the selectable marker, enabling the lettuce to be resistant to spectinomycin. The resulting construct was transformed into *Agrobacterium tumefaciens* strain EHA105 via electroporation, and positive colonies were verified by PCR. For lettuce transformation, five‐day‐old germinated lettuce cotyledons were immersed in the Agrobacterium suspension and incubated at room temperature for 30 min. After infection, the Agrobacterium suspension was removed, and the explants were transferred to co‐culture medium for two days of dark co‐culture at 23°C. Subsequently, explants were transferred to a selection medium supplemented with 50 mg/L spectinomycin and cultivated under light (16 h light/8 h dark photoperiod) at 25°C for four weeks to induce resistant shoots. After that, the resistant shoots were transferred to a spectinomycin‐containing elongation medium and cultivated for 4–6 weeks at 25°C under a 16 h light/8 h dark photoperiod at 5000 lx until rooting was evident. Genomic DNA was then extracted, and the target site was identified through PCR cloning and sequencing. The mutant region from the T_1_ seedling genome was directly cloned using the Plant Direct PCR Kit (Vazyme, China, PD105), according to the manufacturer's instructions, and sequenced to determine whether they were homozygous. T_1_ seeds were obtained from confirmed mutant plants and germinated. The homozygous T_1_ lettuce with gene editing were identified, and T_2_ seeds were harvested.

The homozygous *CRISPR‐lsf3′h* plants, 2 wild lettuce accessions (GWAS‐w35 and GWAS‐w43) and 2 lettuce cultivars belonging to the modern group (ZY and SW17K848), were sown and, one month after transplanting, grown in a plant factory that enables the cultivation of lettuce in multiple layers with precisely controlled environmental conditions and fertigation. During the cultivation, the environmental conditions were maintained as: lighting quality, white LED; lighting intensity, 200 μmol·m^−2^·s^−1^; lighting period, 16 h lighting with 8 h dark; temperature, 20°C ± 2°C; relative humidity, 60%–70%; nutrition solution, Hoagland nutrient solution. The newly expanded leaves were collected for gene expression and metabolite content detection. All samples were ground into a fine powder in liquid nitrogen. The total RNA was isolated from each sample using FastPure Universal Plant Total RNA Isolation Kit (Vazyme, China, RC411‐01), according to the manufacturer's protocols. The cDNA was generated from 1 μg of total RNA using PrimeScript RT reagent Kit with gDNA Eraser (Takara, Japan, RR047A), according to the manufacturer's instructions. RT‐qPCR and data analyses were performed as previously reported (Ying et al. 2020). Furthermore, the 2^−ΔΔCt^ method was utilized to calculate the expression level of the relevant genes. *LsActin* was considered an internal control for calculating the results.

Metabolites were identified and quantified using LC–MS on an Agilent 1290 Infinity II LC coupled with a 6545 LC‐Qtof platform (Agilent Technologies Inc., CA, USA). The analytical conditions were as described in our previous study (Garcia et al. 2020). The commercial standards of quercetin 3‐glucoside, kaempferol 3‐glucuronide, apigenin 7‐glucuronide and luteolin 7‐glucuronide were purchased from Yuanye Bio (Shanghai Yuanye Bio‐technology, Shanghai, China).

### 2.5 Enzyme Activity of LsF3′H Assay Under In Vitro Conditions

Lettuce LsF3′H gene expression vector pESC‐LEU‐LsF3′H was constructed. Subsequently, the expression vector pESC‐LEU‐LsF3′H and the empty vector pESC‐LEU were transformed into yeast strain WAT11, and WAT11‐pESC‐LEU was utilized as the control.

The transformed strain was inserted into 1 mL of SD‐Leu liquid medium (2% glucose) and incubated at 30°C for 24 h. Subsequently, 100 μL of the yeast suspension was aspirated and transferred into 10 mL of SD‐Leu liquid medium (0.2% glucose) and incubated at 30°C for 24 h. The substrate (e.g., naringenin, kaempferol or apigenin) was subsequently added to the yeast suspension, thereby achieving a final substrate concentration of 1 mM. Furthermore, 20% (w/v) galactose solution was added as an inducer at a ratio of 1:100, and the mixture was shaken at 30°C for 72 h. Then, 1 mL of anhydrous methanol was added to 1 mL of fermentation broth. The extract was subsequently vortexed and shaken for 5 min and centrifuged for 10 min. The supernatant was taken out and filtered through a 0.2‐μm membrane and analysed using the Thermo Scientific UltiMate 3000 UPLC system (Thermo Scientific, USA).

## 3 Results

### 3.1 Lettuce Metabolic Profiles

An Orbitrap LC–MS‐based metabolomics approach was used to profile the metabolites present in 40 lettuce accessions to investigate the metabolic diversity of wild accessions and modern lettuce cultivars. More than 13 000 features were extracted from the negative and positive ion mode analyses using Progenesis QI software by setting appropriate parameters for data preprocessing. The analytical characteristics of the metabolic profiles were evaluated to determine the robustness of LC–MS analysis and sampling. A stable RT for each feature was noted in the LC–MS analysis (i.e., the drift time for the same feature in the lettuce and QC samples was < 0.02 min). Additionally, 79.41% (positive ion mode) and 73.06% (negative ion mode) of the features in the QC samples demonstrated variable coefficients < 30%. Furthermore, clustered scores for the QC samples were noted in the PCA model containing lettuce and QC samples (Figure S2A,B). These results suggest the reliability and reproducibility of the metabolomic data acquired from the LC–MS analysis.

The *m/z*, MS^2^ fragments, RT and isotope distribution of each feature were compared with commercial standards, an in‐house lettuce database, online databases (Metlin, HMDB and Lipid Maps), and the literature for metabolite identification. A total of 237 metabolites were putatively identified (Dataset S2), including 12 carbohydrates, 30 amino acid derivatives, 22 organic acids, 11 nucleotides, 100 phenolic compounds (62 phenolic acids and 38 flavonoids), 19 terpenoids, 7 vitamins, 19 lipids and 17 others. According to the proposed minimum reporting standards for metabolite identification (Sumner et al. 2007), among the 237 compounds, 130 were identified at level 1 (identified compounds) by matching their retention time, accurate mass and tandem MS data against authentic analytical standards; of these, 34 were phenolic compounds. An additional 55 metabolites were putatively annotated at level 2, based on physicochemical properties and/or spectral similarity to Metlin MS/MS library, HMDB MS/MS library or published literature.

### 3.2 Metabolite Variation in Wild Ancestors and Modern Lettuce Cultivars

**FIGURE 1:**
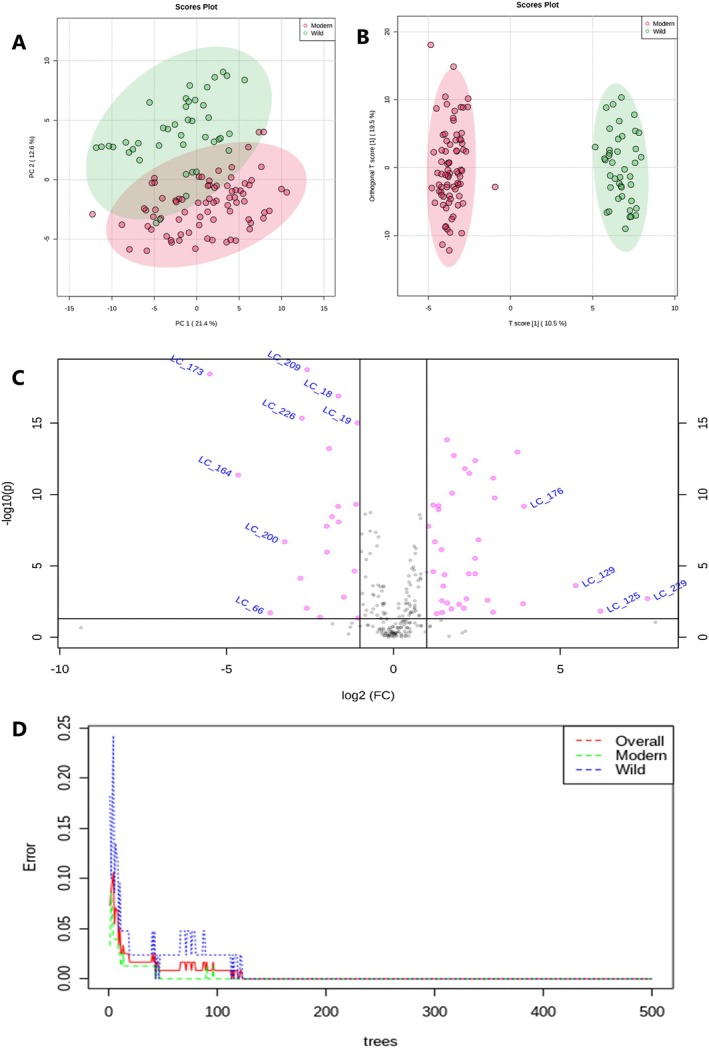
Metabolic differences in wild ancestors and modern lettuce cultivars. (A) PCA analysis, the green and red circle display 95% confidence regions of wild and modern groups; (B) OPLS‐DA analysis; (C) Volcano plot analysis, features with fold‐change > 2 and adjusted *p*‐value < 0.01 were highlighted as red; (D) RF classification, cumulative error rates of RF, the overall, modern and wild class error rate were represented by red, green and blue line, respectively.

The relative abundances of the 237 putatively identified metabolites across 40 lettuce accessions were compared to evaluate the metabolic variations between wild ancestors and modern lettuce cultivars. Unsupervised PCA and K‐means clustering analyses were conducted to assess the differences between the two groups. The score plot of the PCA model showed that PC1 and PC2 accounted for 34.0% of the total variance. Separation of the two groups was noted in the PCA score plot and clustering analysis, although some samples overlapped, suggesting metabolic variation between the wild ancestors and modern lettuce cultivars (Figure 1A; Figure S3).

A supervised OPLS‐DA model was used (Figure 1B; Figure S4) to obtain a direct view of the systematic variations in the metabolomes of wild and modern lettuce samples. The OPLS‐DA model, based on one predictive and one orthogonal component (*R* ^2^ *X* = 0.30, *R* ^2^ *Y* = 0.85, *Q* ^2^ = 0.84), showed an apparent separation between the wild ancestor and modern lettuce cultivars on the abscissa axis (T score [1]). In order to screen the differential metabolites between the wild and modern lettuce groups, we used the combined parameters, including the VIP score of the predictive component with a value > 1.0 (Dataset S3), a fold change > 2 and a *p*‐adjusted value < 0.05 (Figure 1C; Dataset S4), and the top 40 metabolites with the highest mean decrease in accuracy values in RF analysis (Figure 1D; Dataset S5). A Venn diagram was subsequently constructed to identify the biomarkers shared in three independent analyses, which showed that 29 common metabolites were recognized as differential metabolites to distinguish wild ancestors and modern lettuce cultivars (Table 1; Figure S5). These 29 metabolites included 21 polyphenols (four phenolic acid derivatives, three tannins, two lignins and 12 flavonoids), two terpenoids (8‐deoxylactucin and geniposide), two amino acids, two organic acids, one nucleotide derivative and one carbohydrate. Of the 29 metabolites identified, 17 were present in higher concentrations in modern lettuce cultivars, including quercetin glycosides, eriodictyol glucoside, lignins, geniposide and phenolic acids. Contrastingly, tannins, 8‐deoxylactucin, amino acids, organic acids, apigenin glycosides, nucleotide derivatives and phenolic acids were primarily accumulated in wild ancestors.

**TABLE 1:** Differential metabolites in wild ancestor and modern lettuce cultivars.

No.	Name	VIP score (component 1)	Mean decrease accuracy	log_2_(FC_Modern/wild_)	*p* adjusted value
LC_176	Eriodictyol glucoside	1.70	4.40E‐03	3.91	6.82E‐10
LC_185	Eriodictyol glucoside isomer	1.99	9.39E‐03	3.71	1.09E‐13
LC_179	Coniferyl alcohol glucoside(coniferoside) isomer 3	1.77	7.40E‐03	3.03	1.73E‐10
LC_216	Glucose 6‐phosphate	1.86	1.19E‐02	2.99	7.14E‐12
LC_159	Quercetin 3‐O‐(6”‐O‐malonyl)‐glucoside 7‐O‐glucoside isomer	1.50	3.29E‐03	2.54	1.49E‐07
LC_189	Phloridzin	1.94	1.26E‐02	2.45	4.22E‐13
LC_165	Quercetin 3‐(6″‐malonylglucoside)	1.21	2.84E‐03	2.44	3.56E‐05
LC_153	Rutin	1.34	3.95E‐03	2.44	2.96E‐06
LC_138	Quercetin diglucoside isomer	1.91	5.44E‐03	2.27	3.29E‐12
LC_113	Coniferyl alcohol glucoside(coniferoside)	1.86	5.81E‐03	2.14	1.54E‐12
LC_123	Geniposide	2.01	6.83E‐03	1.81	1.88E‐13
LC_169	Quercetin 3‐glucoside −6″‐acetate isomer	1.79	3.60E‐03	1.75	8.20E‐11
LC_105	Dihydrocaffeic acid hexose	2.02	9.19E‐03	1.60	1.46E‐14
LC_54	Dihydroxybenzoic acid hexose	1.16	2.77E‐03	1.52	4.20E‐05
LC_156	Quercetin 3‐glucoside	1.76	2.69E‐03	1.35	6.08E‐10
LC_166	Quercetin 3‐glucoside −6″‐acetate	1.69	6.16E‐03	1.34	1.12E‐09
LC_116	Quercetin 3‐O‐(6”‐O‐malonyl)‐glucoside 7‐O‐glucoside	1.72	3.18E‐03	1.19	5.55E‐10
LC_19	Quinic acid	2.11	7.92E‐03	−1.08	9.49E‐16
LC_95	p‐Coumaroyltartaric acid isomer 1	1.72	5.32E‐03	−1.12	4.88E‐10
LC_18	Tartaric acid	2.19	9.53E‐03	−1.64	1.28E‐17
LC_64	Hydroxyphenyllactic acid hexose	1.68	5.15E‐03	−1.66	6.82E‐10
LC_157	Apigenin diglucoside	2.01	1.03E‐02	−1.92	6.09E‐14
LC_168	Di(4‐hydroxyphenylacetyl)‐hexose isomer 1	1.40	9.03E‐03	−1.99	1.09E‐06
LC_208	L‐Serine	1.57	4.62E‐03	−2.00	1.67E‐08
LC_209	L‐Proline	2.23	9.84E‐03	−2.58	1.83E‐19
LC_226	Succinyladenosine	2.12	6.34E‐03	−2.74	4.57E‐16
LC_200	Tri‐4‐hydroxyphenylacetyl glucoside	1.48	9.80E‐03	−3.26	2.05E‐07
LC_164	Di(4‐hydroxyphenylacetyl)‐hexose	1.88	9.79E‐03	−4.65	4.42E‐12
LC_173	8‐deoxylactucin	2.28	1.17E‐02	−5.50	3.63E‐19

### 3.3 Genetic Structure Determination in Wild and Cultivated Lettuce

Forty lettuce accessions were re‐sequenced to investigate the complex genomic variations resulting from lettuce domestication. Sequencing analysis generated clean reads with 5× average depth and 85.63% median coverage for each sample (Dataset S6). Paired‐end reads were mapped against the assembled lettuce genome (Reyes‐Chin‐Wo et al. 2017), and a final set of 614 576 high‐quality single‐nucleotide polymorphisms (SNPs) was developed following SNP calling and filtration. These genomic variations represent novel resources for lettuce breeding and biological research. We performed a joint analysis with an SNP dataset from the 440 lettuce accessions previously reported by Wei et al. (2021) to evaluate the genetic representativeness of the selected 40 lettuce accessions. We extracted shared SNPs and applied LD pruning (*r* ^2^ < 0.2), given that both datasets were aligned to the same reference genome. Based on the remaining 117 751 high‐quality SNPs, we constructed a maximum‐likelihood (ML) phylogenetic tree including all 480 accessions using FastTree and conducted population structure analysis using ADMIXTURE (Figure S6). Forty accessions were identified at the tips of the phylogenetic tree to visualize their genetic distribution. These 40 accessions were broadly distributed across various phylogenetic branches and spanned the major genetic backgrounds of the entire population, indicating their representativeness and suitability for downstream analyses.

**FIGURE 2:**
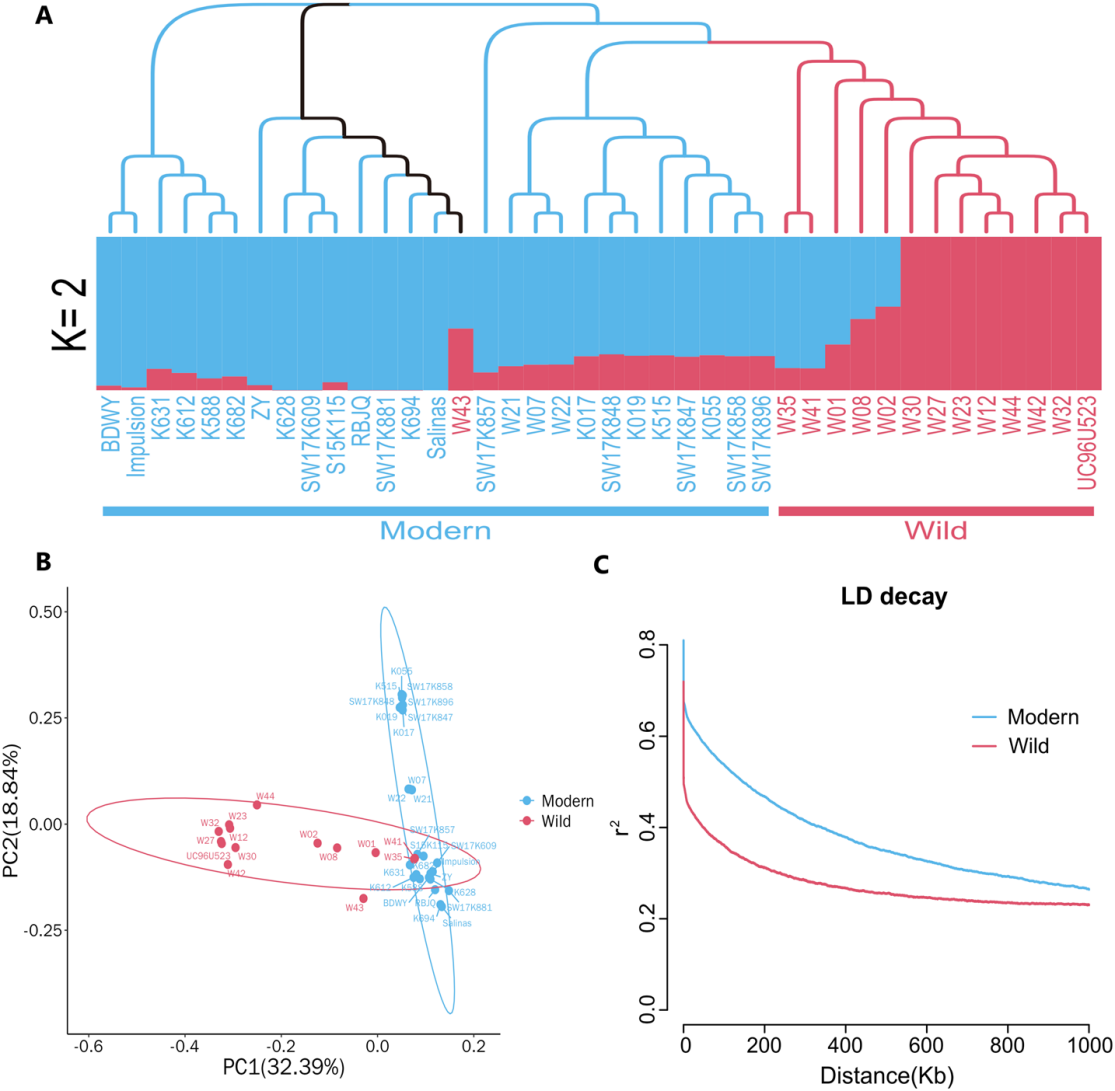
Determination of the genetic structure and genomic variations of wild ancestors and modern lettuce cultivars. (A) ADMIXTURE analysis (*K* = 2); each bar represents one individual, and the length of the coloured bar represents the proportion of the lettuce genome inherited from each ancestral population. (B) PCA of the 40 accessions of wild ancestors and modern lettuce cultivars. PC1 and PC2 explained 32.39% and 18.84% of the variance, respectively. (C) LD decay of wild ancestors and modern lettuce cultivars measured by *r* ^2^.

A range of analytical techniques was employed, including phylogenetic relationships, ADMIXTURE and PCA analyses, to examine the genetic structure of the 40 lettuce accessions. The constructed ML tree showed two distinct groups of wild and cultivated lettuce (Figure 2A). This result was supported by PCA (Figure 2B) and ADMIXTURE analyses when *K* = 2 (Figure 2A). The LD in the wild group decayed to half of its maximum value (*r* ^2^ = 0.3787) at a 61.4 kb physical distance, which was considerably shorter than that of the modern group (285.7 kb, *r* ^2^ = 0.4151) (Figure 2C).

### 3.4 Selection Regions in the Cultivar Lettuce

**FIGURE 3:**
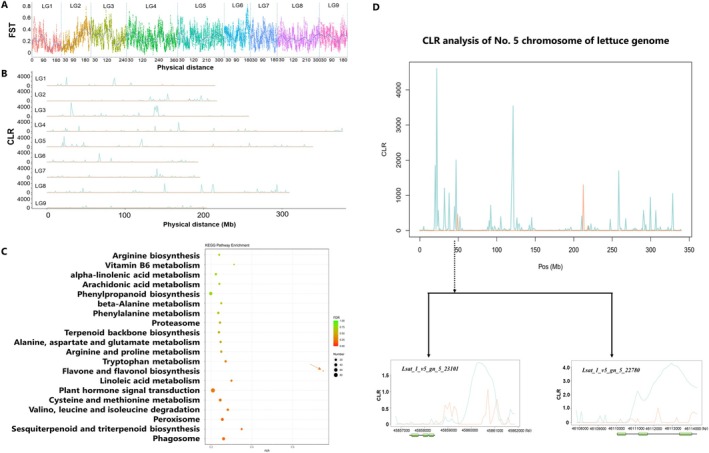
Selection signal analysis screened genes under natural and human selection. (A) FST method; (B) CLR method; (C) KEGG enrichment of genes screened by FST and CLR method; (D) CLR analysis of No. 5 chromosome of lettuce genome, modern cultivars with green line and wild ancestors with orange line; whole genomic sequence of *Lsat_1_v5_gn_5_22780* was subjected to selection, while the promoter region of *Lsat_1_v5_gn_5_23101* was subjected to selection.

Genetic differentiation of the 40 lettuce accessions was evaluated by calculating pairwise values of the fixation index (F_ST_) and the CLR. The F_ST_ values revealed multiple genomic regions with strong differentiation signals distributed across chromosomes 1–9, highlighting potential loci that underwent significant selection in the lettuce cultivar (Figure 3A). The presence of distinct peaks suggests that selective pressures were not evenly distributed but rather concentrated in specific genomic intervals, possibly corresponding to genes involved in key agronomic or adaptive traits. Consistently, the CLR analysis identified widespread signals of selective sweeps across all chromosomes (Figure 3B). Several pronounced CLR peaks were detected, indicating genomic regions that may have experienced recent positive selection during domestication or subsequent breeding improvement. Together, these results suggest that the lettuce genome harbours multiple selection hotspots that contributed to the formation of cultivar‐specific genetic characteristics.

The selected regions detected by merging the F_ST_ and CLR statistics encompassed 6489 genes (Dataset S7). The annotation of these genes facilitated the exploration of genes linked to domestication. Selection signals for domesticated genes were enriched in multiple metabolic pathways involved in the biosynthesis of flavonoids, lignins, terpenes, phenolic acids, organic acids, amino acids and nucleotides (Figure 3C; Dataset S8). Thus, the selection of these candidate genes would have driven the metabolic divergence between wild and cultivated lettuce. Notably, the flavone and flavonol biosynthesis pathways were highly enriched. Two genes (*Lsat_1_v5_gn_5_22780* and *Lsat_1_v5_gn_5_23101*) involved in flavonoid biosynthesis were identified as potential subjects for domestication (Figure 3D) that encode flavonoid 3′‐hydroxylase (F3*′*H). Additionally, analysis of the whole genomic sequence of *Lsat_1_v5_gn_5_22780* suggested that it was subjected to selection. Whereas the promoter region of *Lsat_1_v5_gn_5_23101* was subjected to selection (Figure S7). F3′H is involved in flavonoid synthesis and catalyses hydroxylation at the 3′ position of flavonoids (e.g., kaempferol to quercetin) (Schoenbohm et al. 2000; Cao et al. 2024). Thus, the selection of the LsF3′H enzyme under human selection could potentially lead to the accumulation of glycosylated flavones and flavonols in the metabolite profile of modern lettuce cultivars.

### 3.5 Selection on LsF3′H Contributes to Differences in Flavonol Metabolism Between Wild and Modern Lettuce

We checked the expression levels and patterns of these two *F3*′*H* genes from a previously acquired dataset to verify their function (Xiang et al. 2025). *Lsat_1_v5_gn_5_22780* showed low expression levels in leaves and other organs, whereas *Lsat_1_v5_gn_5_23101* showed high expression levels in the leaves and receptacles (Dataset S9). Subsequently, these two genes were cloned, and their expression levels in leaf samples from two wild lettuce ancestors (GWAS‐w35 and GWAS‐w43) and two modern lettuce accessions (ZY and SW17K848) were determined. However, only the *Lsat_1_v5_gn_5_23101* gene was successfully cloned, whereas the *Lsat_1_v5_gn_5_22780* gene failed to be cloned because of its low expression level in lettuce leaf samples. Subsequently, concentrations of the glycosylated quercetins in the five accessions were quantitatively analysed. The expression levels of *Lsat_1_v5_gn_5_23101* in freshly expanded leaves of a wild lettuce ancestor and four modern lettuce accessions were measured and compared with previously tested metabolomic data. The expression levels of *Lsat_1_v5_gn_5_23101* and the levels of glycosylated quercetin in wild lettuce leaves were lower than those in modern lettuce leaves (Figure S8).

**FIGURE 4:**
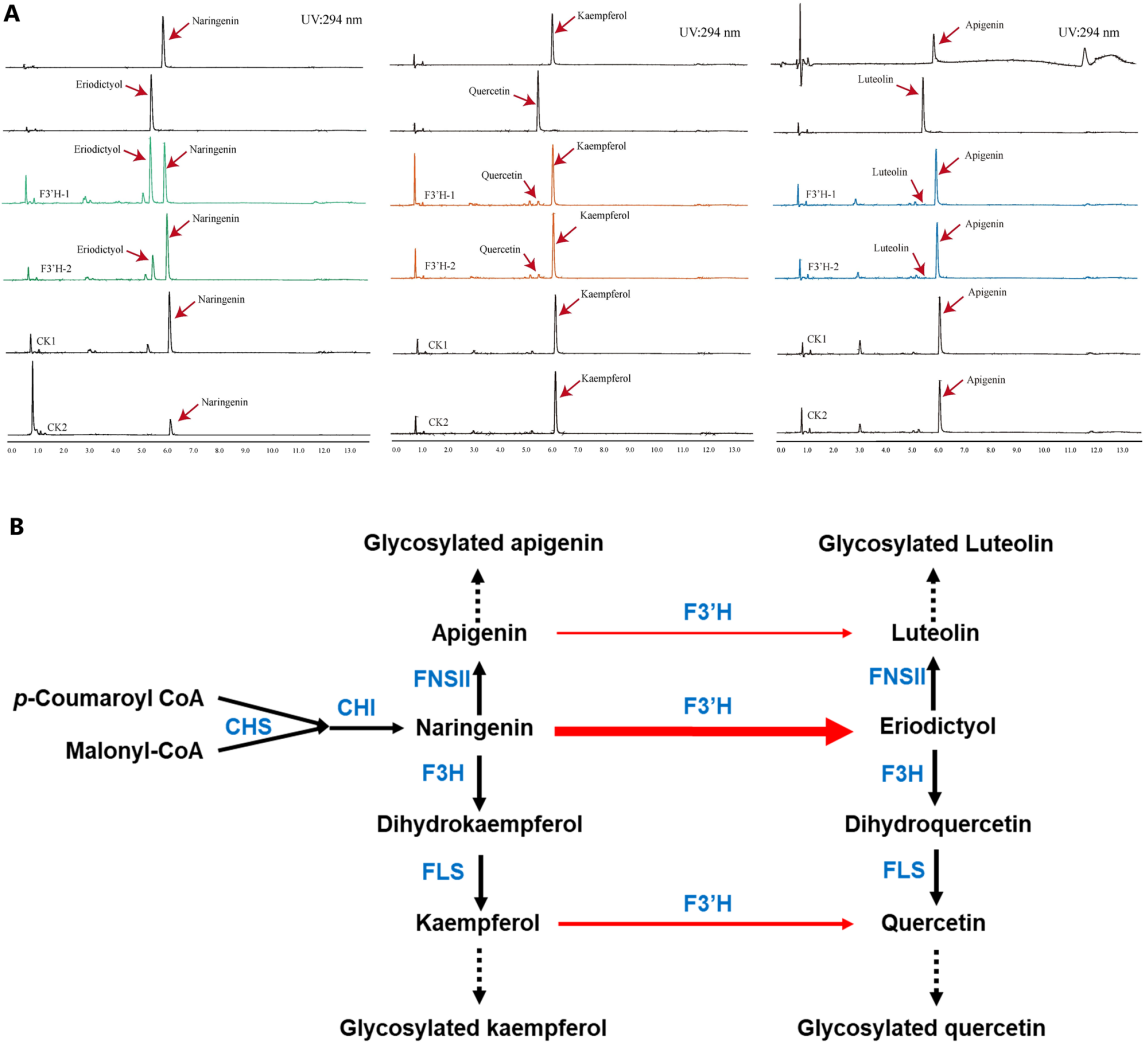
Enzymatic activity assessment of LsF3*′*H in vitro. (A) LsF3′h catalysed enzymatic reaction; results indicated that LsF3*′*H could catalyse the production of eriodictyol, quercetin or apigenin from naringenin, kaempferol or luteolin respectively; (B) proposed enzymatic reaction pathway catalysed by LsF3′H.

The enzyme activity of Lsat_1_v5_gn_5_23101 encoded F3′H in lettuce (LsF3′H) was determined using an in vitro evaluation to identify the enzymatic function of Lsat_1_v5_gn_5_23101 in lettuce. Subsequently, the substrate of the LsF3′H catalysed enzymatic reaction (e.g., naringenin, kaempferol or apigenin) was added to the yeast suspension, and the respective enzymatic products were determined via UPLC. LsF3′H could catalyse eriodictyol, quercetin or luteolin production from naringenin, kaempferol or apigenin, respectively (Figure 4A); while the peak areas of quercetin and luteolin were generally smaller than that of eriodictyol, which suggested that LsF3′H had a higher capacity to catalyse the reaction when naringenin was used as the substrate. Then, an enzymatic reaction pathway was constructed that was catalysed by LsF3′H (Figure 4B).

Then, we analysed the nucleotide sequences in the 2 kb upstream region of the *LsF3*′*H* gene in 40 accessions (Figures S9 and S10; Dataset S10). The sequences of the modern accessions exhibited a high degree of consistency, with almost no variations detected, and no alterations in the *cis*‐regulatory elements were observed. In contrast, for several individuals in the wild accessions (e.g., W08, W12, W44, W02, W42 and UC96U523), the sequences exhibited significant divergence from those of the modern accessions, while the remaining individuals demonstrated substantial consistency with them. Moreover, the *cis*‐regulatory elements are non‐coding DNA sequences that have the capacity to recruit transcription factors and affect gene expression. In order to investigate variations in the promoter of *LsF3*′*H* and the potential mechanism, a *cis*‐regulatory element analysis was conducted on the promoters of all 40 accessions (Figure S11). The results indicated that variants in the *LsF3*′*H* promoter had a significant impact on the formation of various *cis*‐regulatory elements, including the chs‐CMA1a, the O2‐site, the TCT‐motif and the TGA‐element. These components have been demonstrated to affect the binding of transcription factors, such as O_2_ transport protein, TGA and MYB, and could be regulated by environmental factors such as light, thereby potentially influencing the expression of *LsF3′H*. These results suggested that these variants in *LsF3′H* promoter had been fixed in the modern accessions after domestication; whereas in the wild group, this region might be in the dynamic evolution stage of the domestication process. Also, we analysed the variations within the coding sequence region of *LsF3′H* in the wild and modern lettuce groups and predicted the severity of mutations. The results suggested that the coding sequence region contains 6 synonymous mutations and 1 non‐synonymous mutation (Dataset S11). Given that the prediction tools trained on plant data are more suitable for vegetable genomics, we employed the plant‐specific Plant Protein Variation Effect Detector (PPVED) platform to evaluate this non‐synonymous substitution (Gou et al. 2022). The prediction result indicated that this mutation has a potential functional impact (Figure S12). Then, we compared the sequences of the LsF3*′*H enzyme proteins and found no differences between the wild and modern lettuce groups.

**FIGURE 5:**
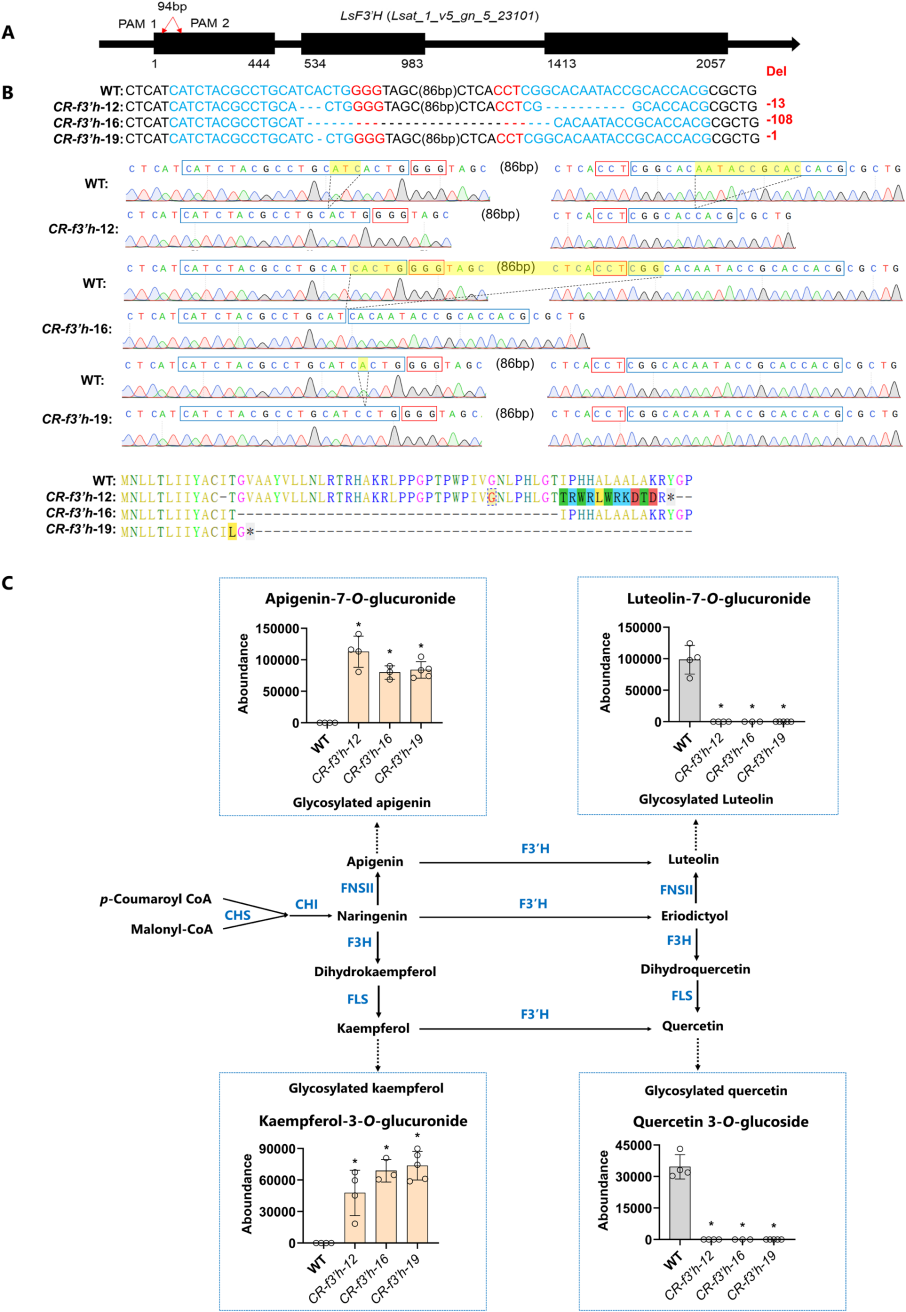
*LsF3′H* is a key gene for the biosynthesis of quercetin derivatives in lettuce. (A) Schematic representation of two sgRNAs (highlighted in red arrows) for targeting mutations in the *LsF3′H* (*Lsat_1_v5_gn_5_23101*) coding sequence. (B) Sequence‐based genotyping of CRISPR/Cas9‐*lsf3′h* (*CR‐f3′h*) plants with positive editing. The target sites were indicated in blue letters, and the PAM sites were highlighted in red. Del represents the number of deletions in the coding sequence in base pairs (bp). (C) The contents of flavonoid compounds in *CR‐f3'h* plant leaves were detected by UPLC‐MS. Data were shown as means ± SD (*n* = 3 – 5). *Indicates the significant difference compared to the wild type (WT) ‘jingyan yidali’ (*p < 0.05).

Multiple homozygous mutant lines of *LsF3′H* were obtained from a green looseleaf lettuce cultivar ‘jingyan yidali,’ and three of them were cultured to 1 month old for metabolite detection (Figure 5A–D), to further study the gene function of *Lsat_1_v5_gn_5_23101* (*LsF3′H*) in lettuce. Compared to the wild type, the *LsF3′H* knockout mutants demonstrated a substantial decrease in the contents of luteolin (e.g., luteolin 7‐glucoside) and quercetin derivatives (e.g., quercetin 3‐glucoside) while simultaneously showing a considerable increase in the contents of apigenin (e.g., apigenin 7‐glucuronide) and kaempferol derivatives (e.g., kaempferol 3‐glucuronide). Thus, Lsat_1_v5_gn_5_23101 plays a crucial role in the catalysis of hydroxylation at the 3′‐position of flavonoids (e.g., apigenin to luteolin and kaempferol to quercetin). Together with the knockout experiments and enzymatic analyses, these results provide further evidence that the promoter region of *LsF3′H* has been a key target of selection during domestication, driving the differential expression and consequent metabolic divergence between wild ancestors and cultivated lettuce.

## 4 Discussion

Lettuce is one of the most widely consumed leafy vegetables globally and has substantial commercial value and nutritional significance because of the presence of health‐promoting compounds. Metabolomics methods can qualitatively and quantitatively comprehensively analyse a wide range of metabolite classes and are powerful tools for revealing changed metabolite expression in different lettuce varieties. Consequently, a comprehensive understanding of the natural variation and genetic basis of plant metabolism will aid in the utilization of these variations to improve the nutritional quality of crops, thereby ensuring food security and quality. However, the accurate identification of metabolites, such as flavonoids, which is the most time‐consuming and challenging step, is hindered by the presence of different structural isomers and the limited availability of MS^2^ tag‐based metabolite libraries to date (Creek et al. 2014). In the present study, the high‐resolution accurate mass system embedded in the UHPLC‐QE Orbitrap MS facilitated the simultaneous detection and quantification of metabolites, especially secondary metabolites, in wild and modern lettuce cultivars. A total of 237 metabolites were putatively identified, of which 130 were cross‐validated based on chemical standards and several spectral data (identification level 1), of which 12 were glycosylated by quercetin, apigenin and luteolin. This has resulted in a considerable extension of our understanding regarding the diversity of primary and secondary metabolites in lettuce and has substantially facilitated the exploration of how domestication influences lettuce quality.

Plant metabolome comprises a wide variety of metabolites with varying degrees of polarity. Currently, there is no single extraction method and analytical platform can offer full coverage of the plant metabolome. Thus, the selection of the extraction solvent significantly restricts the observation of the metabolome. In this study, several amino acids, organic acids and secondary metabolites (e.g., phenolic acids, flavonols, anthocyanins and terpenes) were found to be soluble and capable of detection in LC–MS by using aqueous methanol or acidified aqueous methanol as solvent. Whereas, it has been demonstrated that several classes of metabolites, including carotenoids, phytosterols, fat‐soluble vitamins, fatty acids and their esters, exhibit limited solubility in aqueous methanol and acidified aqueous methanol solutions (Vuckovic 2012). Further plant metabolomics studies could employ a combination of analytical platforms (e.g., GC–MS and LC–MS) and extraction methods, as well as several advanced chromatographic separation technologies (e.g., ion mobility‐derived collision cross section), which enable a wide range of metabolite classes and m/z scan ranges, and facilitate accurate characterization of structural isomers (Lanucara et al. 2014; Yang, Feng, et al. 2018). Moreover, metabolic dynamics exhibit a high degree of correlation with coupled environmental factors, such as light, temperature, humidity, fertilizers and CO_2_. In this study, the environmental conditions in the greenhouse could not be fully controlled due to the lack of smart sensors and the ineffectiveness of the coordination of heating, cooling and ventilation equipment. Given that metabolite accumulation in lettuce is strongly influenced by environmental factors, multi‐environment trials are essential for evaluating the stability of metabolic traits and understanding genotype‐by‐environment (G × E) interactions. Future studies should therefore evaluate these lettuce accessions across multiple locations and growing seasons, or alternatively in controlled‐environment agricultural systems that allow precise regulation of environmental factors (e.g., plant factories), in order to obtain more reliable and robust insights into metabolic variation (Zhang et al. 2022; Chadwick et al. 2024).

Various analytical techniques, including volcano plots, VIP values and random forest analysis, were employed to determine the robustness of the results of differential metabolites between wild ancestors and modern cultivars. Consequently, 17 metabolites, including 11 flavonoids (eight quercetin glycosides, two eriodictyol glucosides and one dihydrochalcone), two lignins, two phenolic acids, one iridoid and one carbohydrate, demonstrated higher concentrations in modern lettuce cultivars, whereas 12 metabolites were primarily accumulated in wild ancestors, including three tannins, two amino acids, two organic acids, two phenolic acids, one apigenin glycoside, one nucleotide derivative and one sesquiterpene lactone. To the best of our knowledge, all of these 29 metabolites have been reported to be present in lettuce (Abu‐Reidah et al. 2013; Viacava et al. 2018; Yang, Wei, et al. 2018; Yang, Feng, et al. 2018; Xiang et al. 2025). A comprehensive understanding of the genetic basis underlying the biosynthesis of these metabolites in lettuce, along with their regulatory networks, which are influenced by the surrounding environment, will not only facilitate understanding of how plants evolved functionally diverse metabolites as a key adaptive strategy to the environment but also aid in breeding biofortified lettuce cultivars through biotech‐based breeding techniques (Weng et al. 2021; Zhang et al. 2022).

Flavour quality is crucial in the consumer experience of lettuce consumption (Chadwick et al. 2016). In this study, compounds related to astringency (Medel‐Marabolí et al. 2017), bitterness (Frank et al. 2006; Seo et al. 2009) and sourness (Kader 2008; Xu et al. 2023) showed higher accumulation levels in wild ancestors than in modern lettuce cultivars, which suggested that the lettuce domestication process led to a decrease in certain compounds that have a detrimental effect on taste. In the context of consumer purchasing decisions, bitterness was the most significant factor influencing lettuce purchase (Park and Lee 2006). In the present study, 8‐deoxylactucin, a sesquiterpene lactone that contributes to lettuce bitterness (Sessa et al. 2000; Seo et al. 2009) and quinic acid, which exhibits an aspirin‐like bitter taste with a recognition threshold of 10 ppm in food (Frank et al. 2006), were found to accumulate at significantly higher levels in wild lettuce ancestors than in modern cultivars. This differential accumulation may result in stronger bitterness in wild ancestors than in modern lettuce cultivars. In addition, it is widely accepted that, in general, hydrolyzable tannins are perceived as bitter and astringent at high concentrations (Medel‐Marabolí et al. 2017). In this study, three hydrolyzable tannins that might contribute to astringency accumulated to a greater extent in wild ancestors than in lettuce cultivars, including di(4‐hydroxyphenylacetyl)‐hexose, di(4‐hydroxyphenylacetyl)‐hexose isomer 1 and tri‐4‐hydroxyphenylacetyl glucoside. Quinic acid and tartaric acid also contribute to the sour substances in fruits and vegetables (Kader 2008; Xu et al. 2023). In this study, higher organic acid concentrations, including quinic acid and tartaric acid, were noted in the wild species. The reduction in quinic acid levels noted in modern lettuce cultivars was consistent with the results of a previous study, which suggested that human selection resulted in a reduction in quinic acid in cultivated lettuce for quality improvement (Zhang et al. 2020). The decrease in certain compounds that substantially influence flavour is also a crucial feature in the domestication of other horticultural crops. For example, the levels of tannins and organic acids in apples were reduced during domestication (Liao et al. 2021; Lin et al. 2023). Moreover, flavonoids, especially flavonols, were suggested as the main metabolic differences between wild and modern cultivars in this study, with modern lettuce varieties containing higher levels of glycosylated quercetin derivatives than wild species. Herein, *LsF3′H* selection resulted in the accumulation of a large number of glycosylated quercetin derivatives, as was verified by gene function evidence and in vitro enzyme activity evaluation. Previous studies suggested that the astringency threshold of quercetin is 33.1 μM in vegetables such as lettuce, whereas that of glycosylated quercetin derivative rutin and quercetin 3‐glucoside are 0.001 μM and 0.65 μM in horticultural plants (e.g., tea), respectively (Huang and Xu 2020). From a flavour perspective, quercetin glycosylation can mitigate its bitterness. The selection of new lettuce varieties with high levels of glycosylated quercetin derivatives has enabled the preservation of the desired flavour profile while eliminating undesirable bitterness. From the perspective of plant resistance, wild ancestors of lettuce tend to accumulate sesquiterpene lactones, such as 8‐deoxylactucin, a differential metabolite identified in the present study. This class of metabolites confers resistance to both biotic and abiotic stresses, yet it also represents the primary source of bitterness in lettuce (Rees and Harborne 1985; Seo et al. 2009; Padilla‐Gonzalez et al. 2016). In modern lettuce breeding practices, cultivars with a low‐bitterness or non‐bitter flavour trait at commercial maturity are preferentially selected, which may reduce sesquiterpene lactone content and consequently compromise resistance to biotic and abiotic stresses. The dihydroxy B‐ring‐substituted flavonoid (quercetin 3‐glycoside derivatives) possesses a superior capacity to scavenge ROS compared to the vast majority of other flavonoids (Brunetti et al. 2013). Additionally, the accumulation of quercetin glycoside derivatives enhanced lettuce resistance to abiotic and biotic conditions (Jing et al. 2024; Yang et al. 2024), offering a potential alternative to reducing sesquiterpene lactones. Thus, lettuce domestication was hypothesized to lead to the reduction of certain compounds that have adverse effects on taste, including tannins, organic acids and sesquiterpene lactones, and would also lead to the induction of certain compounds that contribute to plant resistance, including glycosylated flavonoids and lignins.

Integrating metabolomics with high‐throughput genomic data can improve our understanding of the genetic underpinnings of natural metabolic variation in lettuce, especially with respect to the domestication effects (Zhang et al. 2020). In the domestication process, the selection of a specific gene invariably leads to a decrease in the nucleotide diversity of the flanking regions of the gene and is known as a selective sweep (Liu et al. 2019). Selective sweeps are valuable indicators of target genes under natural selection or domestication (Pavlidis et al. 2013). In this study, 6489 genes were situated in regions with selective sweeps. Compared to the 2178 genes identified by Zhang et al. (2017) within putative selective sweep regions, our study detected 6489 genes under potential selection. This discrepancy is likely due to the differences in the analytical framework and biological focus of the two studies. Zhang et al. (2017) used a combination of cross‐population composite likelihood ratio (XP‐CLR) and π ratio analyses of *L. serriola* and cultivated lettuce to detect genomic regions associated with the early domestication process. While our analysis used BayeScan and CLR methods to identify selective sweeps mainly within cultivated lettuce accessions, thereby capturing both domestication‐ and breeding‐related selection signals. The larger number of accessions and the use of complementary detection approaches in our study may have also increased the resolution and sensitivity for detecting selection signatures. Subsequently, gene annotation and metabolic pathway enrichment analysis were integrated to identify flavonoids, lignin, terpenoids, phenolic acids, organic acids, amino acids and nucleotide biosynthesis as being subject to natural selection or domestication in lettuce. These results could reflect the breeders' preferences, especially in recent decades, given that the majority of artificial selection in lettuce is due to the deliberate cultivation of specific traits, including quality improvement and plant resistance (Damerum et al. 2020; Hassan et al. 2021). Moreover, the candidate gene *LsF3′H* situated in the regions of selective sweeps was screened using selection signal analysis and verified by gene function study and enzyme activity assessment. This showed that this gene plays a crucial role in catalysing flavonoid hydroxylation at the 3′‐position, which is a result of natural variation and artificial selection in the domestication process of lettuce. The results of this study provide a valuable dataset for the systematic investigation of lettuce domestication to improve its quality.

In conclusion, a comprehensive integrated metabolomics and high‐throughput genomics analysis was conducted to detect variations in lettuce metabolites in a diverse lettuce population comprising 40 accessions of wild and modern lettuce cultivars. Using an LC–MS‐based metabolomics approach, 237 metabolites from 40 accessions of wild and modern lettuce cultivars were putatively identified, and 130 metabolites were identified as level 1. The 29 metabolites associated with lettuce quality were found to differ between wild ancestors and modern cultivars, with flavonol glycoside changes further linked to *LsF3′H* through functional validation. Gene function study and enzyme activity assessment verified that LsF3*′*H was involved in catalysing flavonoid hydroxylation at the 3′‐position of flavonol biosynthesis, which was a result of artificial selection in the domestication process of lettuce. These findings provide a comprehensive view of the metabolite diversity, metabolic pathway differences and potential quality improvements associated with flavonol biosynthesis in lettuce.

## Author Contributions

X.Y., S.W. and B.L. designed the study. R.D. and X.Y. performed the LC–MS analysis. G.J., L.Z., T.H. and J.P. analysed the metabolomics data. S.W., G.H., C.S. and B.L. performed genome resequencing and analysed the data. Y.W. and F.Z. performed the in vitro enzymatic analyses. G.J., L.Z. and T.H. performed the molecular analyses. X.Y., S.W. and B.L. wrote the first draft. V.H.E.C., F.T., L.L., Y.L., B.S. and Q.Y. contributed to the discussion and reviewed the paper, providing comments on the final version.

## Funding

This work was supported by National Natural Science Foundation of China (32202471; X.Y.), Project of Fund for Stable Support to Agricultural Sci‐Tech Renovation (xjnkywdzc‐2022001‐6; B.L.); Sichuan Science and Technology Program (2026YFHZ0321; X.Y. and V.H.); the earmarked fund for Sichuan Innovation Team Program of CARS (sccxtd‐2024‐22; X.Y.), the Initial Fund for Bringing in Talent at Xinjiang Academy of Agricultural Sciences (2022001; B. L.), National Key Laboratory for Germplasm Innovation & Utilization of Horticultural Crops (Horti‐KF‐2024‐06; X.Y.), Local Financial Funds of the National Agricultural Science and Technology Center, Chengdu (NASC2024KY20; B.S.), the Agricultural Science and Technology Innovation Program of CAAS (ASTIP‐IUA‐2026002 and S2024005; Y.L. and L.Z.), and the Elite Youth Program of the Chinese Academy of Agricultural Sciences (X.Y). The funders had no role in study design, data collection and analysis, decision to publish or preparation of the manuscript.

## Conflicts of Interest

The authors declare no conflicts of interest.

## Supporting information


**Figure S1:** Characteristics of 40 accessions used in this study.
**Figure S2:** PCA score plot of lettuce and QC samples.
**Figure S3:** The clustering result of 40 accessions used in this study.
**Figure S4:** Permutation tests of the OPLS‐DA model.
**Figure S5:** Venn diagram of the differential metabolites between wild ancestors and modern lettuce cultivars.
**Figure S6:** Evaluation of the genetic representativeness of the 40 selected lettuce accessions.
**Figure S7:** Selection signals analysis of *LsF3*′*H* (*Lsat_1_v5_gn_5_23101*) between wild and modern lettuce groups.
**Figure S8:** The expression levels of *LsF3*′*H* and concentrations of quercetin glycoside derivatives in selected wild and modern cultivars of lettuce.
**Figure S9:** Phylogenetic analysis of the promoter sequences of *LsF3*′*H* in 40 accessions.
**Figure S10:** Promoter sequences analysis of *LsF3*′*H* between wild and modern lettuce groups.
**Figure S11:** *Cis*‐acting element analysis of LsF3′H promoter between wild and modern lettuce groups.
**Figure S12:** PPVED‐based prediction indicates that the single non‐synonymous mutation in *LsF3*′*H* has a potential functional impact.

**Dataset: S1.** Genotypes and major morphological features of the lettuce accessions used in this study.
**Dataset: S2** Lettuce metabolites putatively identified by LC–MS.
**Dataset: S3** The VIP scores of metabolites in wild ancestors and modern lettuce cultivars.
**Dataset: S4** The volcano plot analysis of metabolites in wild ancestors and modern lettuce cultivars.
**Dataset: S5** The Mean Decrease Accuracy of metabolites in wild ancestors and modern lettuce cultivars.
**Dataset: S6** Re‐sequencing information of wild ancestors and modern lettuce cultivars.
**Dataset: S7** Candidate genes detected by merging FST and CLR statistics.
**Dataset: S8** KEGG pathway enrichment of candidate genes selected by FST and CLR statistics.
**Dataset: S9** F3′H gene expression levels in different growth stages and organs of the lettuce cultivar ‘Ziguan’.
**Dataset: S10** Cis‐acting element analysis of *LsF3*′*H* promoter between wild and modern lettuce groups.
**Dataset: S11** The variations in CDS region of *LsF3*′*H* in wild and modern lettuce groups.

## Data Availability

The genome resequencing data of lettuce were available in the NCBI online database with bio‐project number PRJNA1127331. The data supporting the findings presented in this study are available within the article and its Supporting Information.
