# Supplementary material for: Integrated Metabolomics and Selection Signal Analysis Provide Insights Into the Selection for Flavonol Biosynthesis Associated With Lettuce Quality Improvement
Source: Plant Biotechnol J. 2026 Jul 13:10.1111/pbi.70716. Online ahead of print. doi: 10.1111/pbi.70716 (PMC13398757; doi:10.1111/pbi.70716)
Supplement: Supplementary file 1 — Figure S1: Characteristics of 40 accessions used in this study. Figure S2: PCA score plot of lettuce and QC samples. Figure S3: The clustering result of 40 accessions used in this study. Figure S4: Permutation tests of the OPLS‐DA model. Figure S5: Venn diagram of the differential metabolites between wild ancestors and modern lettuce cultivars. Figure S6: Evaluation of the genetic representativeness of the 40 selected lettuce accessions. Figure S7: Selection signals analysis of LsF3′H (Lsat_1_v5_gn_5_23101) between wild and modern lettuce groups. Figure S8: The expression levels of LsF3′H and concentrations of quercetin glycoside derivatives in selected wild and modern cultivars of lettuce. Figure S9: Phylogenetic analysis of the promoter sequences of LsF3′H in 40 accessions. Figure S10: Promoter sequences analysis of LsF3′H between wild and modern lettuce groups. Figure S11: Cis‐acting element analysis of LsF3′H promoter between wild and modern lettuce groups. Figure S12: PPVED‐based prediction indicates that the single non‐synonymous mutation in LsF3′H has a potential functional impact. [file PBI-9999-0-s001.docx]

**Supplemental Figure Legends:**

**Supplemental Figure S1** Characteristics of 40 accessions used in this study. A, Phenotypes of the 40 accessions used in the study; B, Phylogenetic analysis of the 40 accessions from our core subset of high-quality lettuce breeding projects. These 40 accessions were broadly distributed across various phylogenetic branches and spanned the major genetic backgrounds of the entire population, indicating their representativeness and suitability for downstream analyses.

**Supplemental Figure S2** PCA score plot of lettuce and QC samples. PCA analysis was conducted using SIMCA-P (version 14.0). A, positive ion mode; B, negative ion mode.

**Supplemental Figure S3** The clustering result of 40 accessions used in this study. The distance measure used Euclidean and clustering algorithm used ward. D. Separation of the two groups was noted in this clustering analysis, although some samples overlapped, suggesting metabolic variation between the wild ancestors and modern lettuce cultivars.

**Supplemental Figure S4** Permutation tests of the OPLS-DA model. Q2= 0.964 (*P*<0.01) and R2Y=0.984 (*P*<0.01) suggested the model had good fitting accuracy.

**Supplemental Figure S5** Venn diagram of the differential metabolites between wild ancestors and modern lettuce cultivars.

**Supplemental Figure S6** Evaluation of the genetic representativeness of the 40 selected lettuce accessions.

**Supplemental Figure S7** Selection signals analysis of *LsF3'H* (*Lsat_1_v5_gn_5_23101*) between wild and modern lettuce groups.

**Supplemental Figure S8** The expression levels of *LsF3’H* and concentrations of quercetin glycoside derivatives in selected wild and modern cultivars of lettuce. Wild group: GWAS-w35 and GWAS-w43; Modern group: Ziya, and SW17K848 (n=3).

**Supplemental Figure S9** Phylogenetic analysis of the promoter sequences of *LsF3’H* in 40 accessions.

**Supplemental Figure S10** Promoter sequences analysis of *LsF3'H* between wild and modern lettuce groups.

**Supplemental Figure S11** *Cis*-acting element analysis of LsF3'H promoter between wild and modern lettuce groups.

**Supplemental Figure S12** PPVED-based prediction indicates that the single non-synonymous mutation in LsF3'H has a potential functional impact.


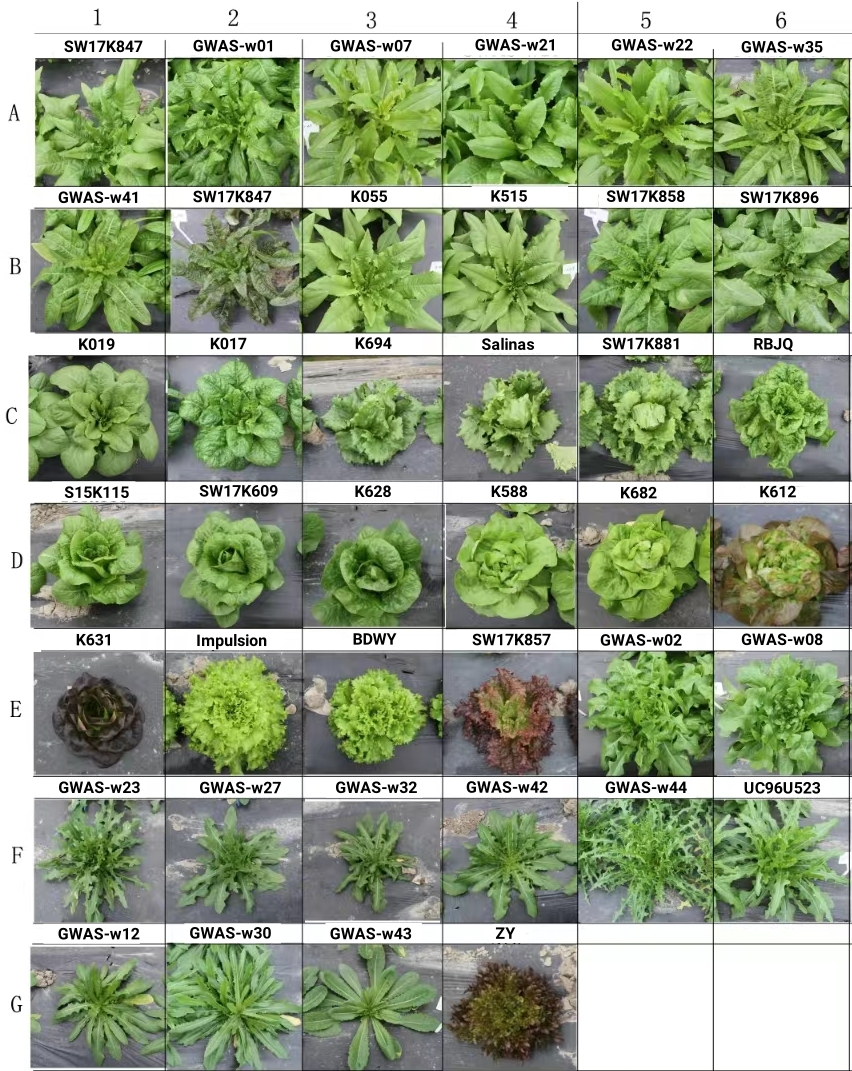
**A**

**B**

**
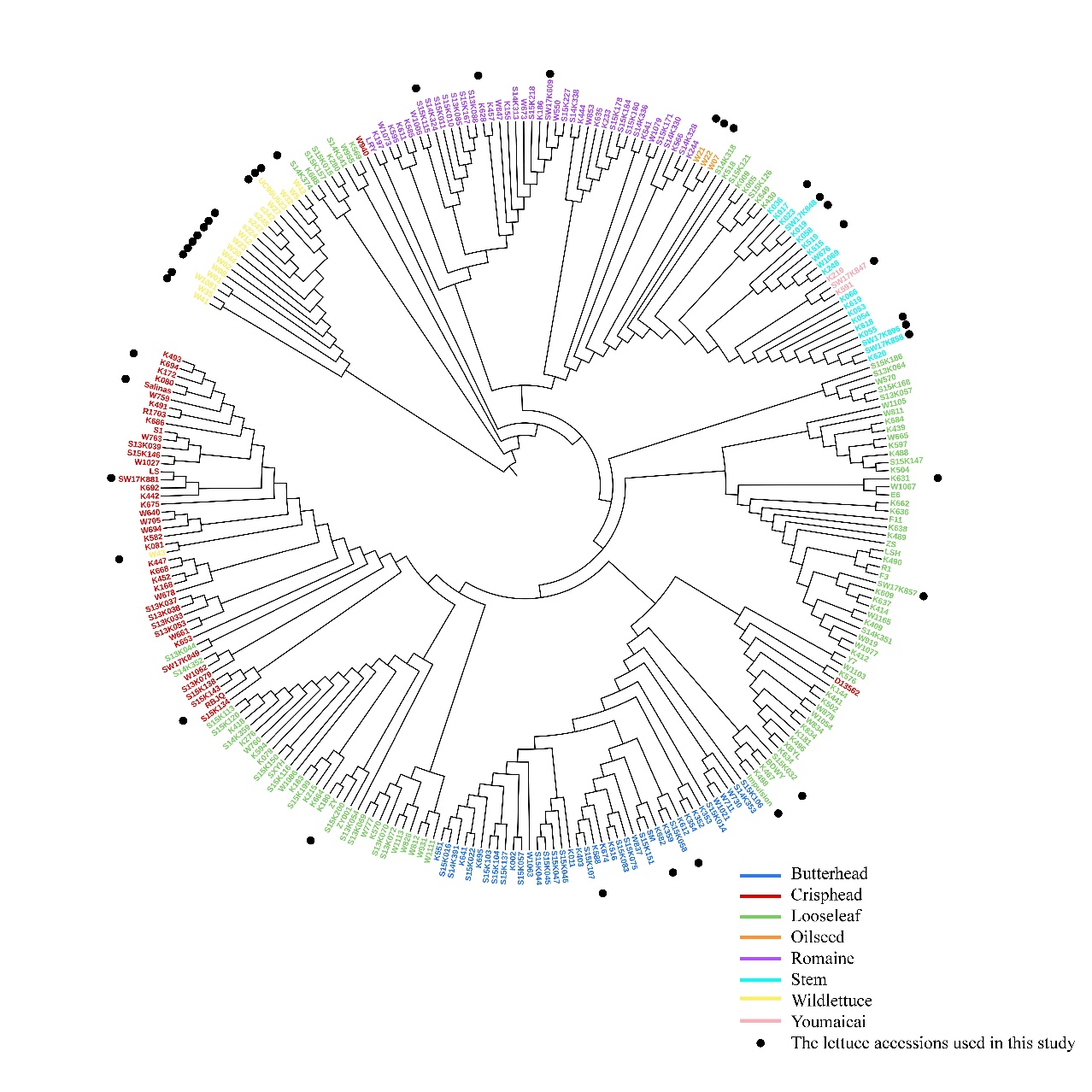
**

**Supplemental Figure S1** Characteristics of 40 accessions used in this study.

A, Phenotypes of the 40 accessions used in the study; B, Phylogenetic analysis of the 40 accessions from our core subset of high-quality lettuce breeding projects. These 40 accessions were broadly distributed across various phylogenetic branches and spanned the major genetic backgrounds of the entire population, indicating their representativeness and suitability for downstream analyses.


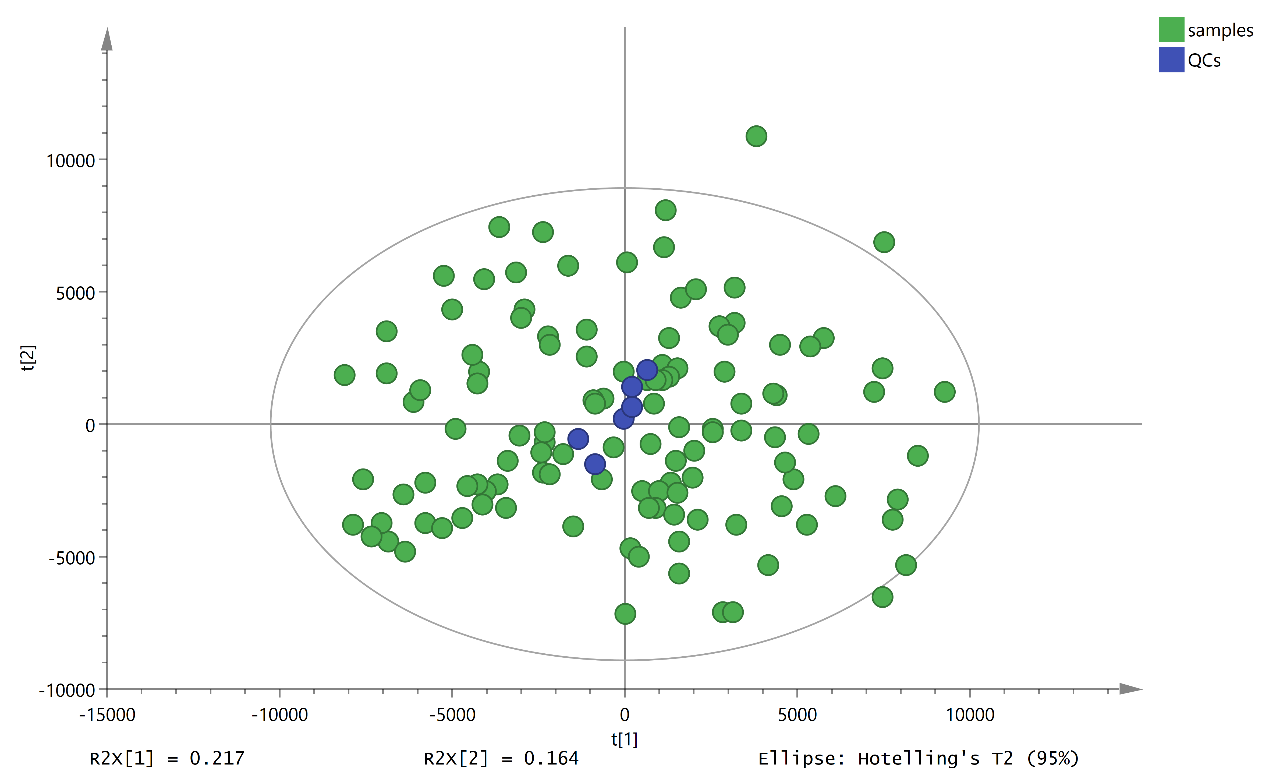


A


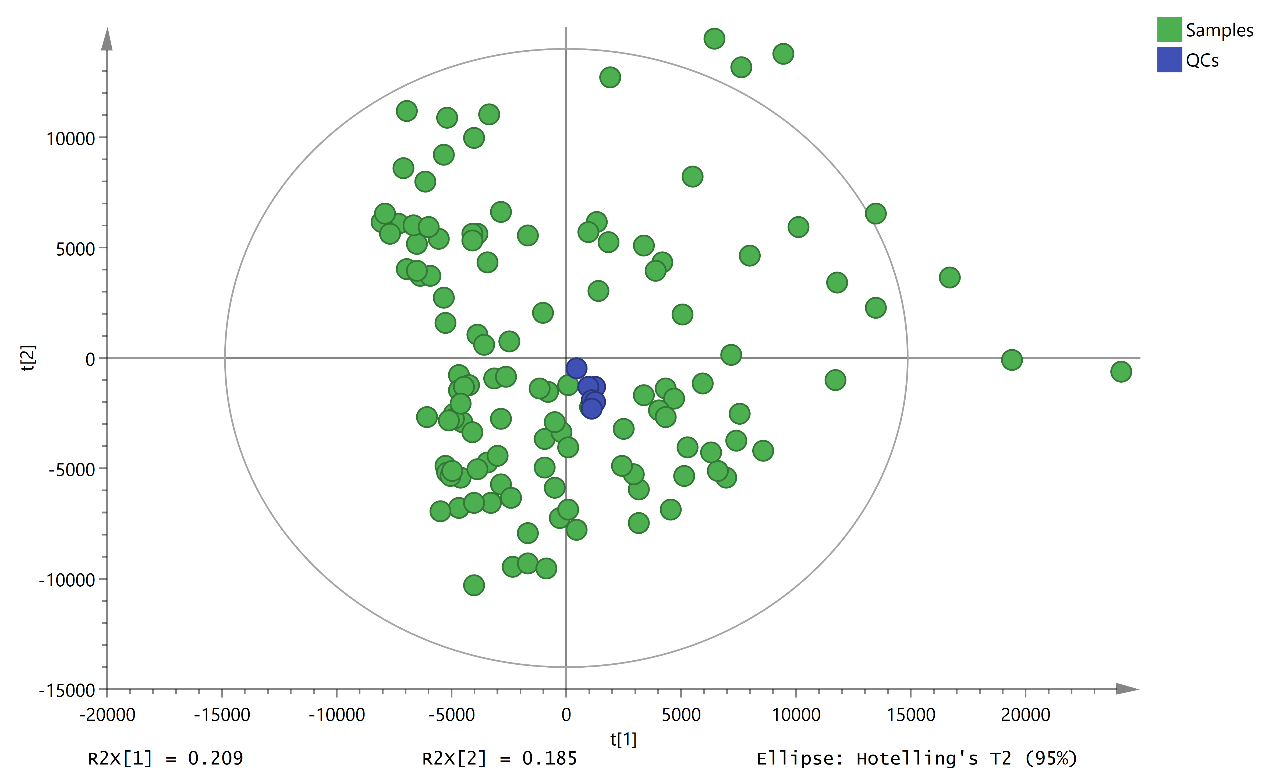


B

**Supplemental Figure S2** PCA score plot of lettuce and QC samples. PCA analysis was conducted using SIMCA-P (version 14.0). A, positive ion mode; B, negative ion mode.


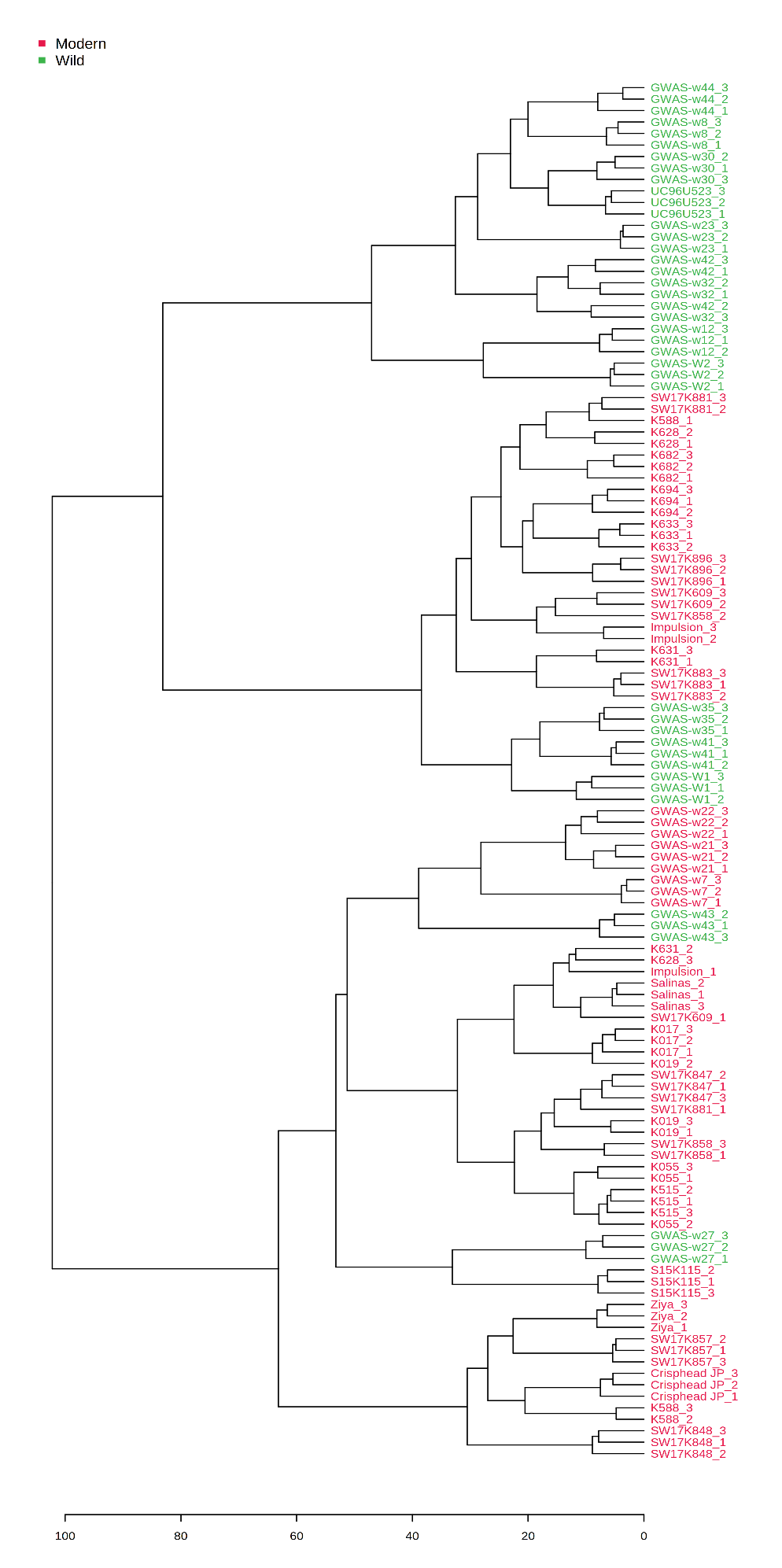


**Supplemental Figure S3** The clustering result of 40 accessions used in this study. The distance measure used Euclidean and clustering algorithm used ward. D. Separation of the two groups was noted in this clustering analysis, although some samples overlapped, suggesting metabolic variation between the wild ancestors and modern lettuce cultivars.


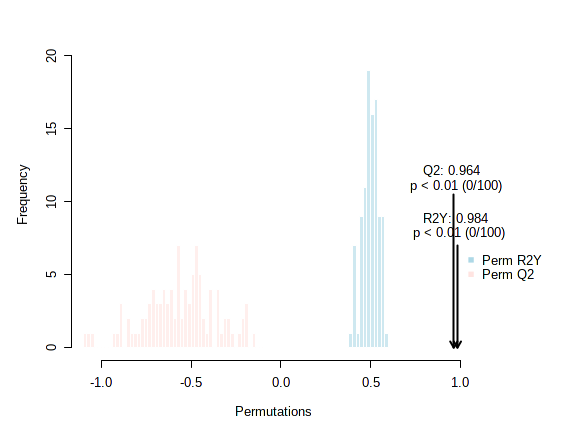


**Supplemental Figure S4** Permutation tests of the OPLS-DA model. Q2= 0.964 (*P*<0.01) and R2Y=0.984 (*P*<0.01) suggested the model had good fitting accuracy.


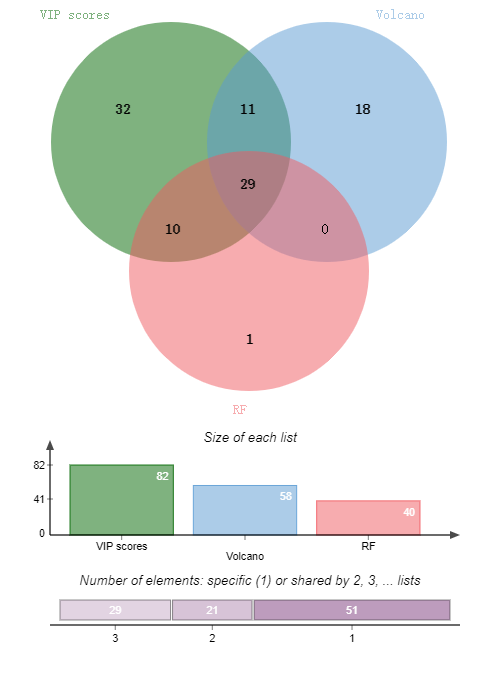


**Supplemental Figure S5** Venn diagram of the differential metabolites between wild ancestors and modern lettuce cultivars.


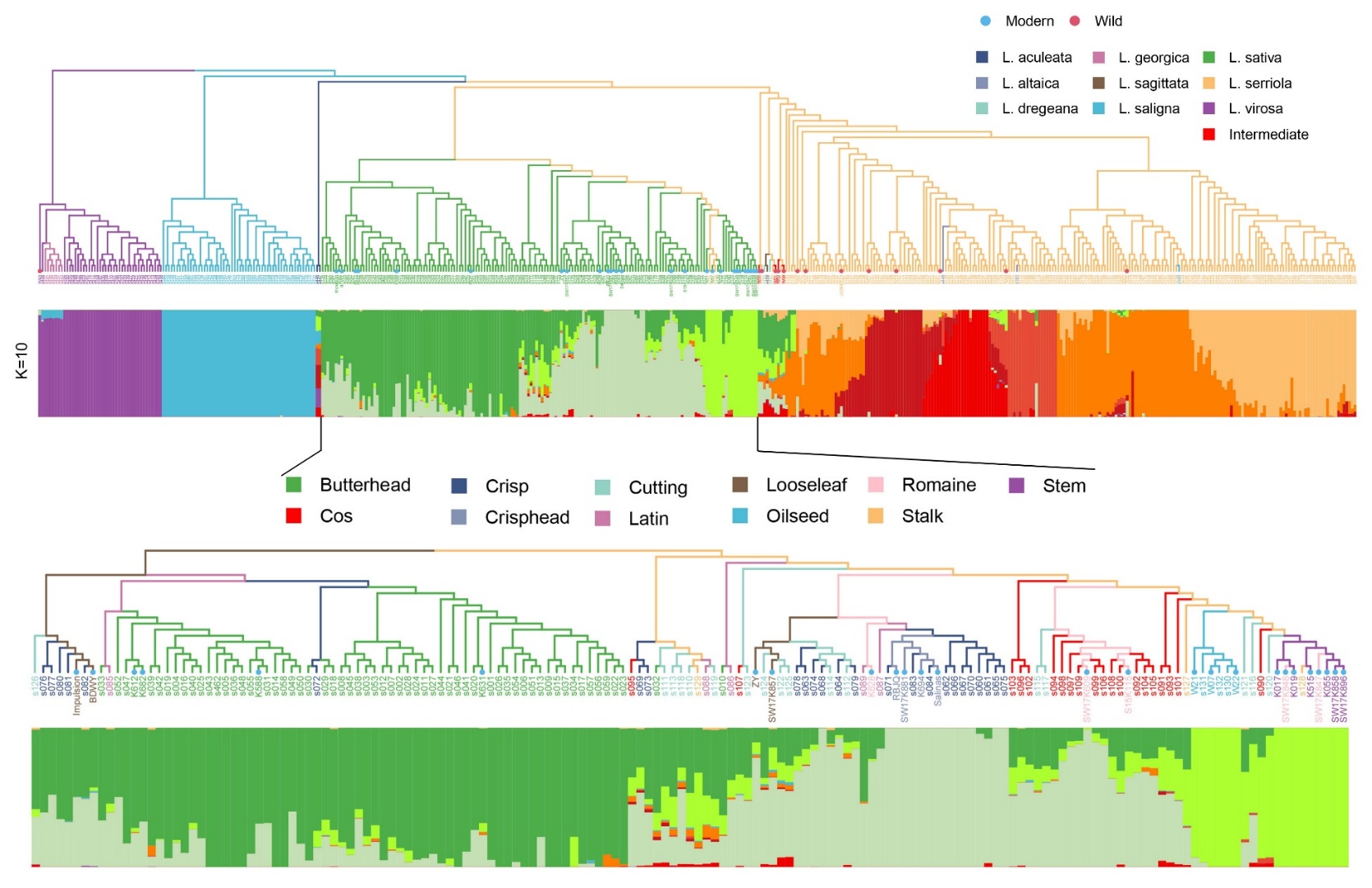


**Supplemental** **Figure S6** Evaluation of the genetic representativeness of the 40 selected lettuce accessions.


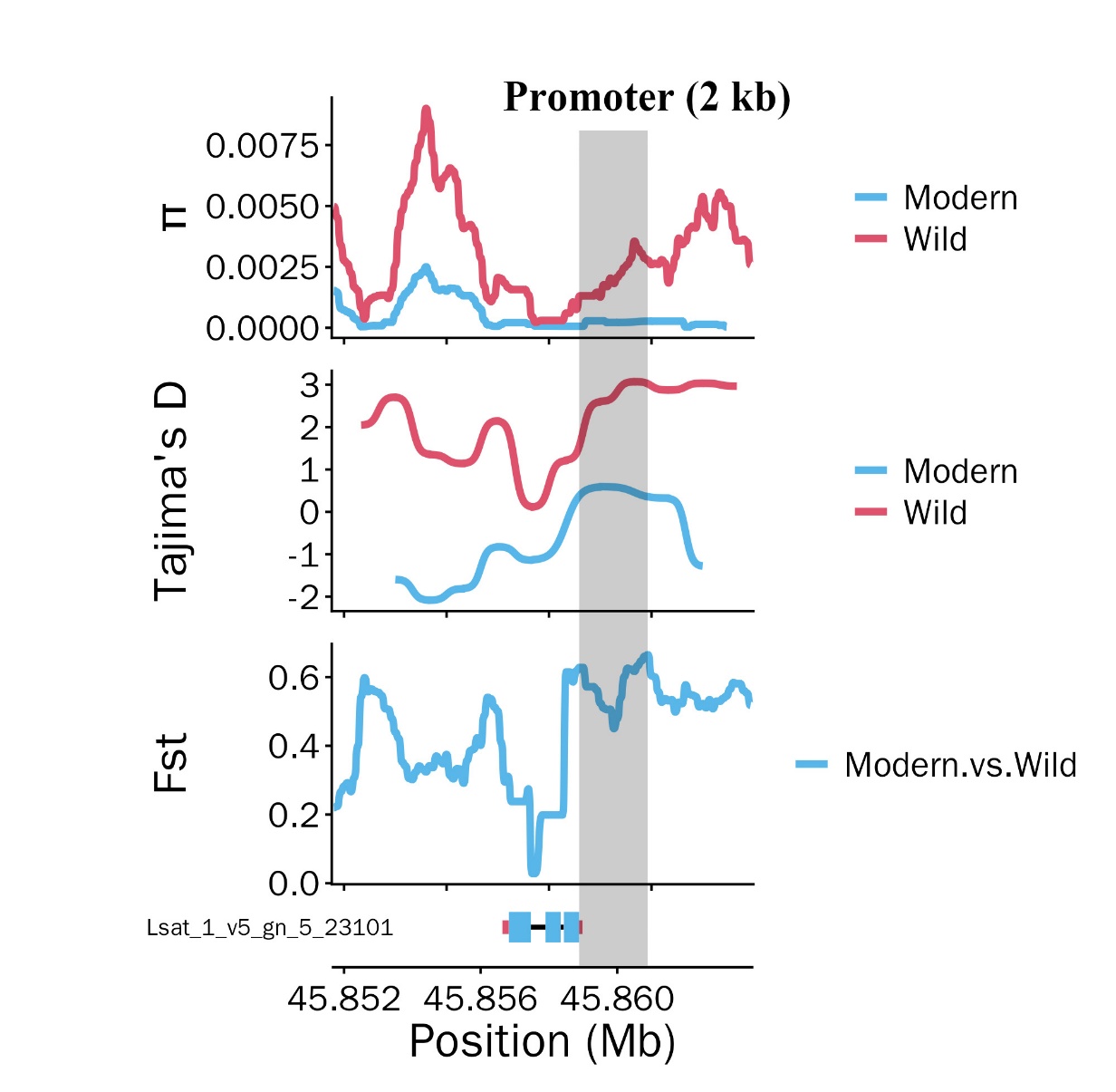


**Supplemental Figure S7** Selection signals analysis of *LsF3'H* (*Lsat_1_v5_gn_5_23101*) between wild and modern lettuce groups.


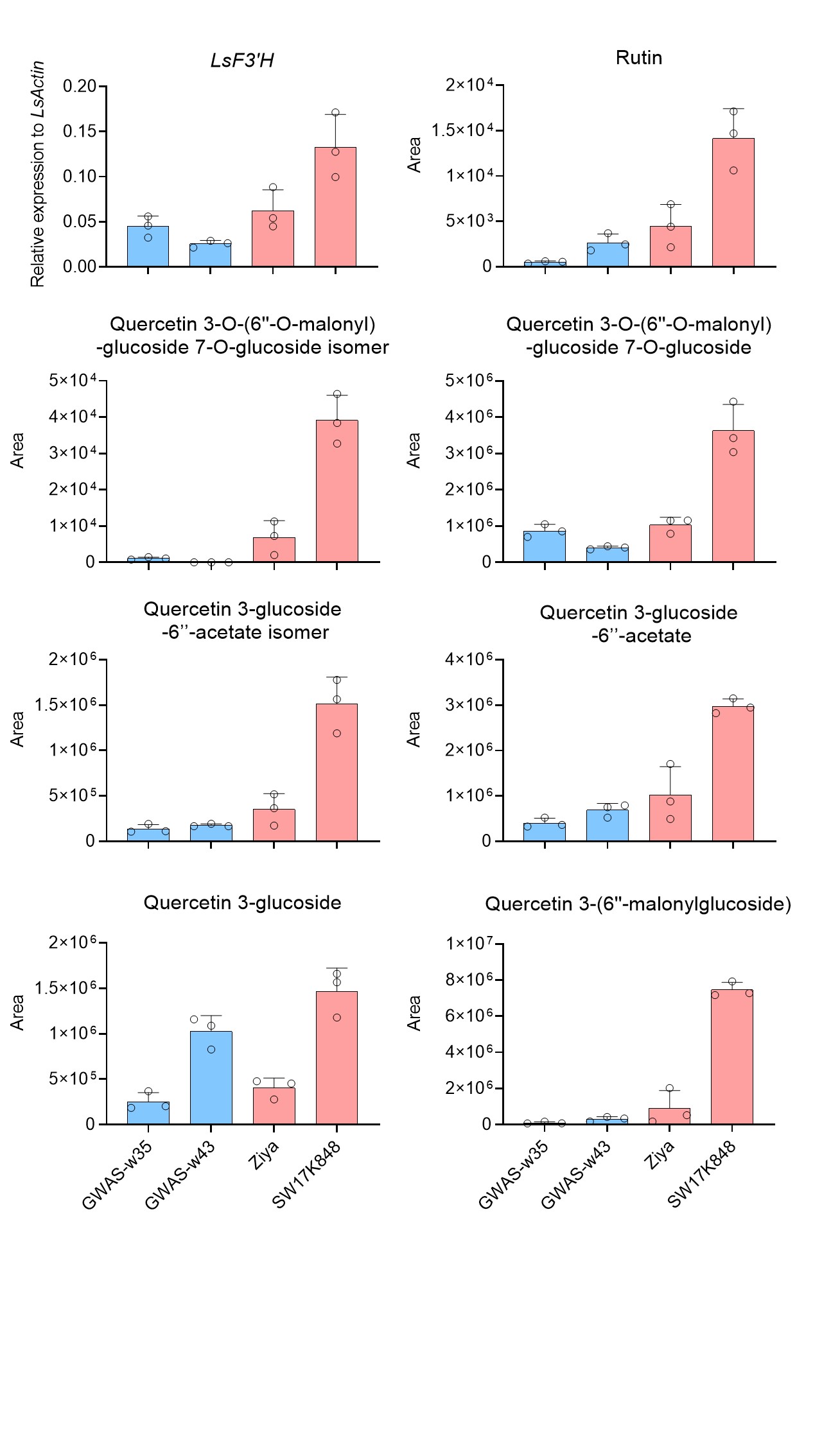


**Supplemental Figure S8** The expression levels of *LsF3’H* and concentrations of quercetin glycoside derivatives in selected wild and modern cultivars of lettuce. Wild group: GWAS-w35 and GWAS-w43; Modern group: ZY (Ziya), and SW17K848 (n=3).

**
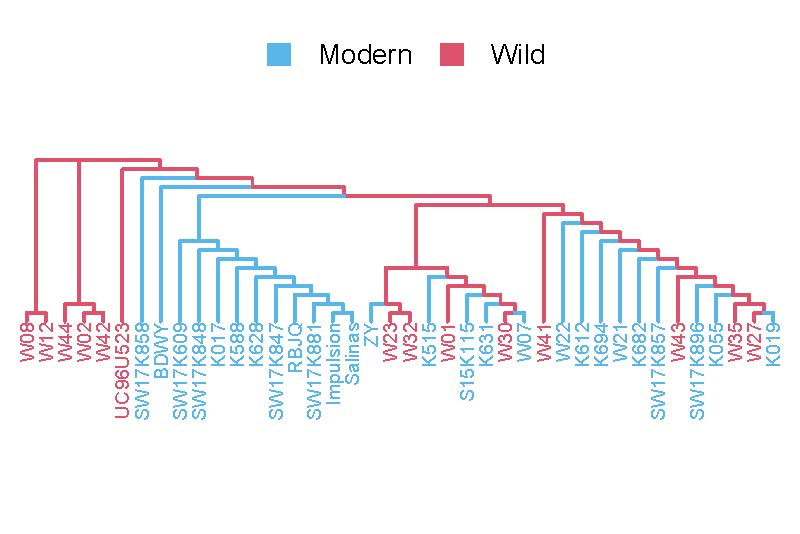
**

**Supplemental Figure S9** Phylogenetic analysis of the promoter sequences of *LsF3’H* in 40 accessions.


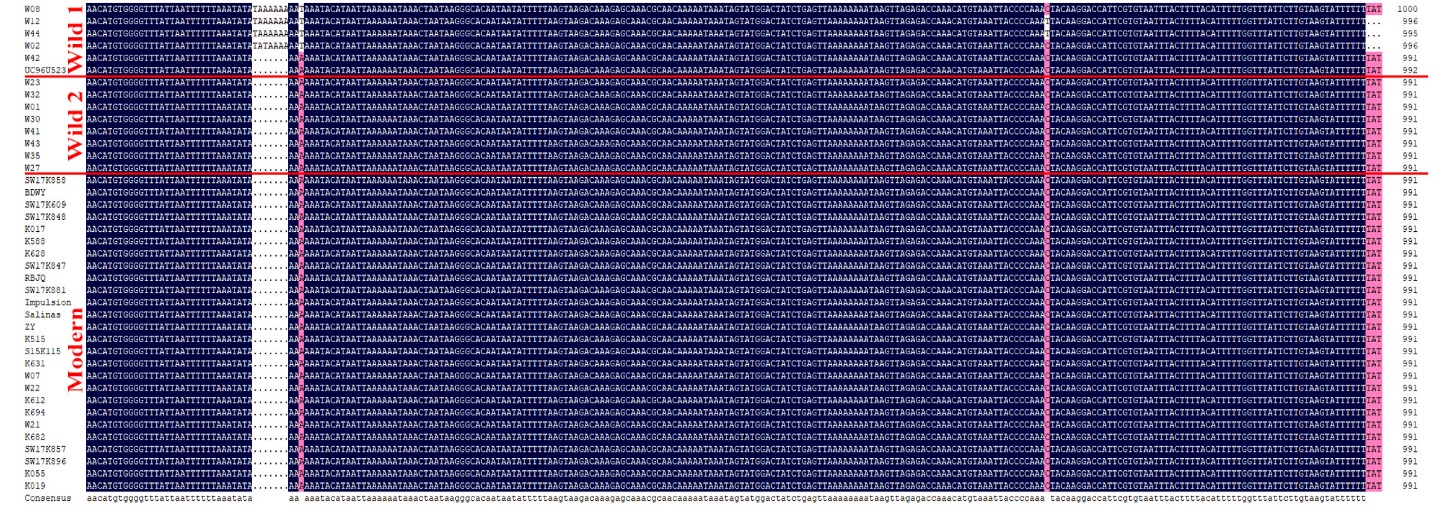

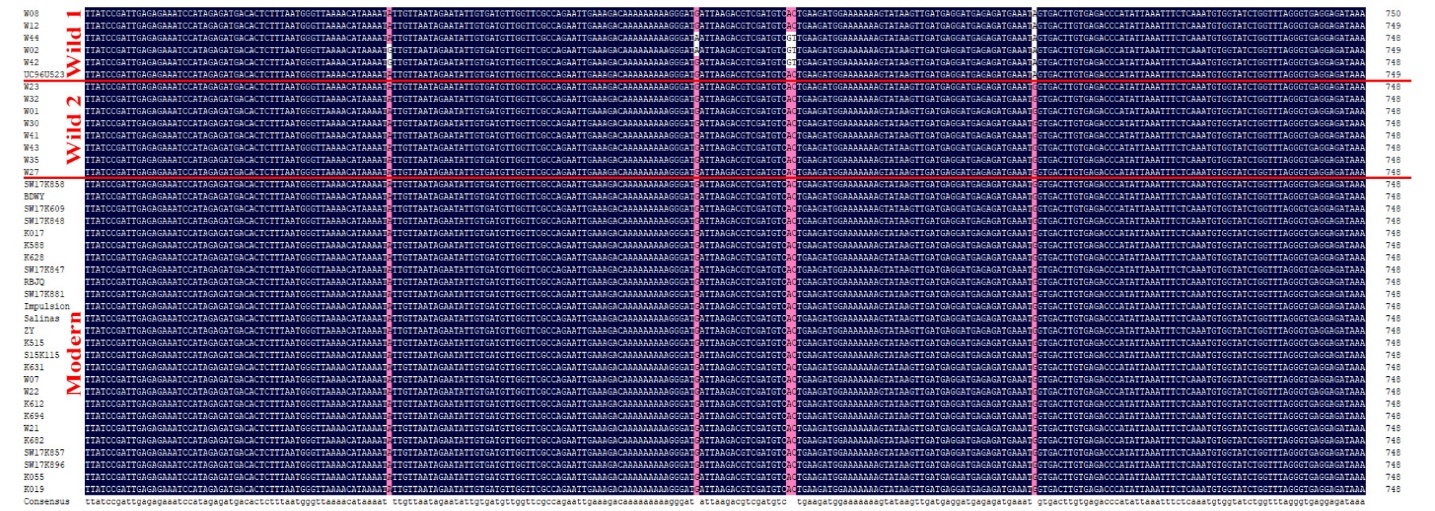

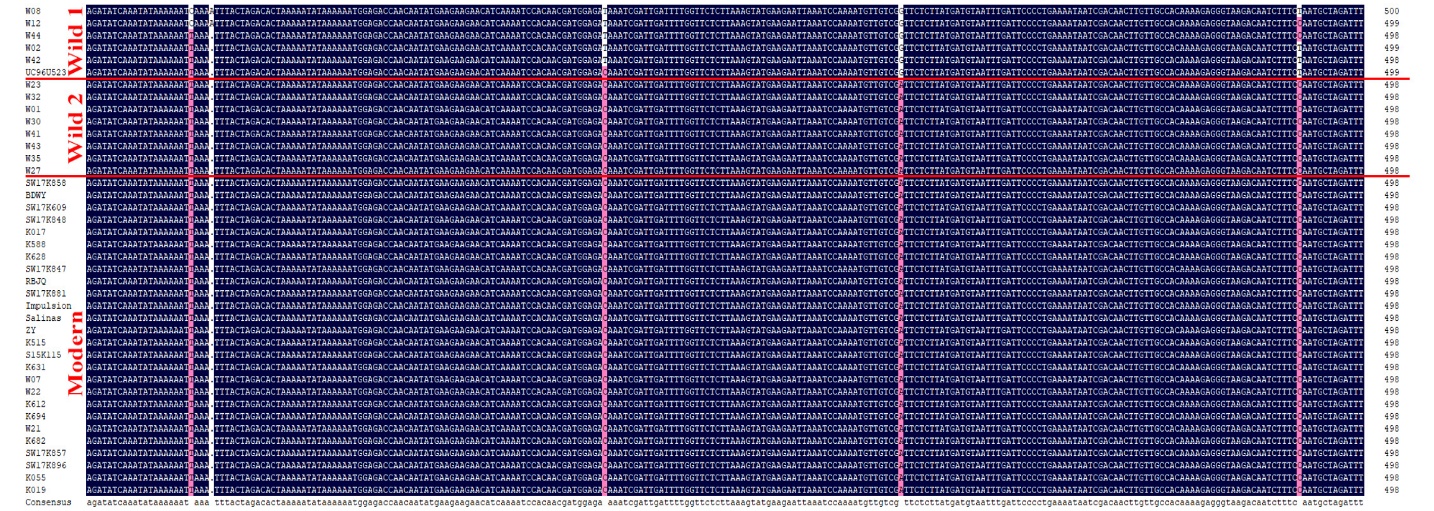

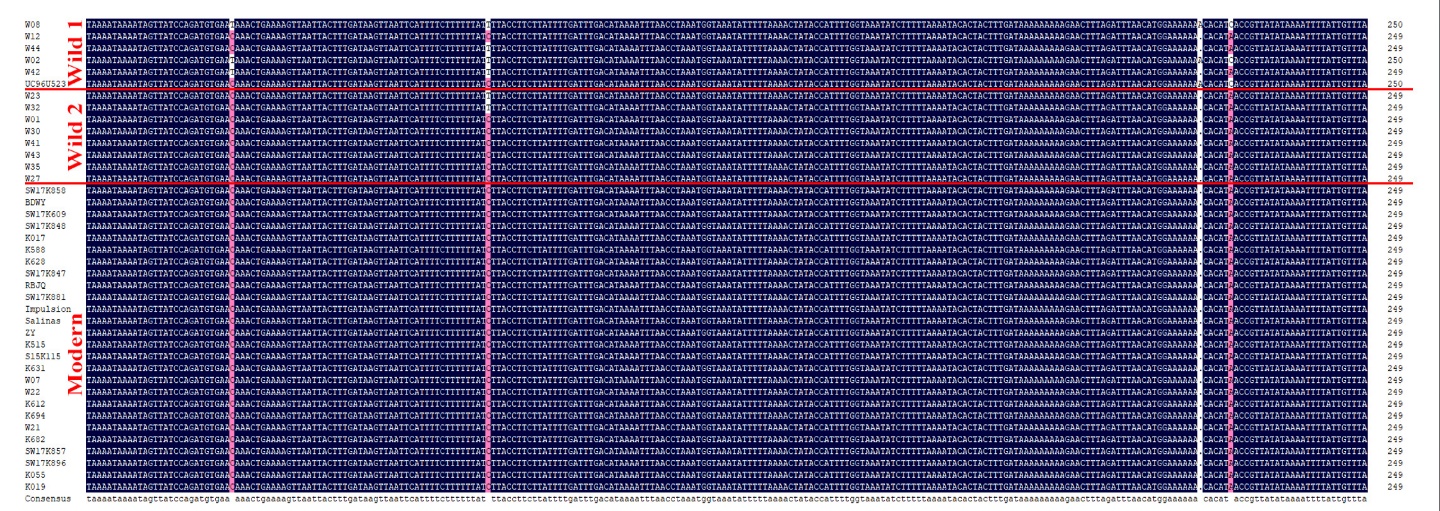


**
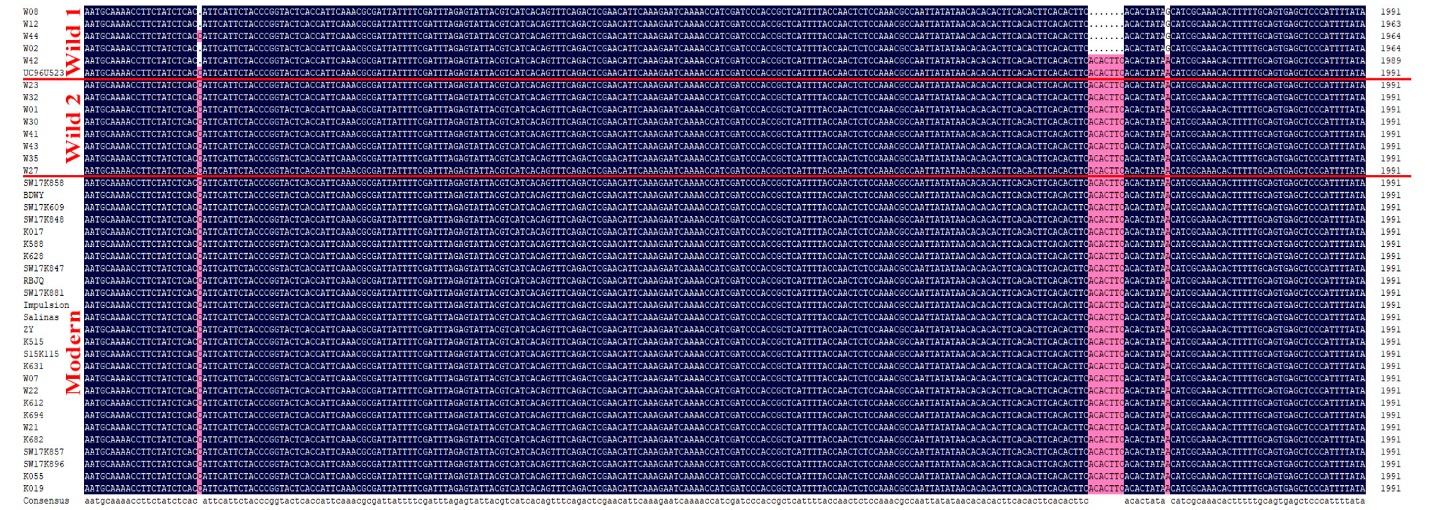

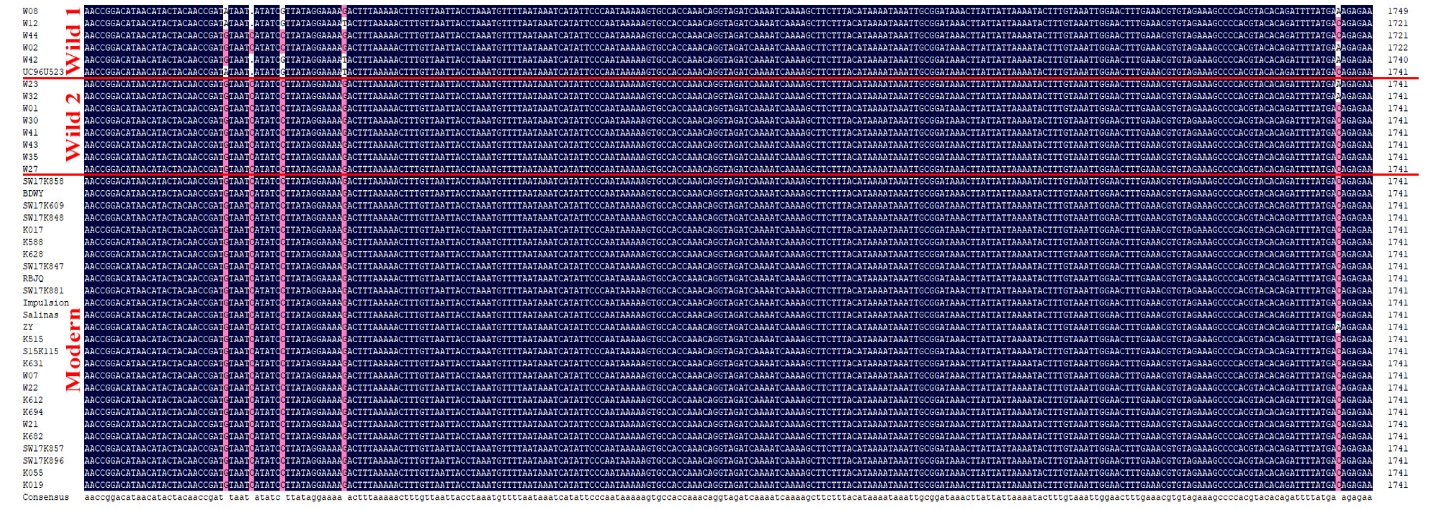
**
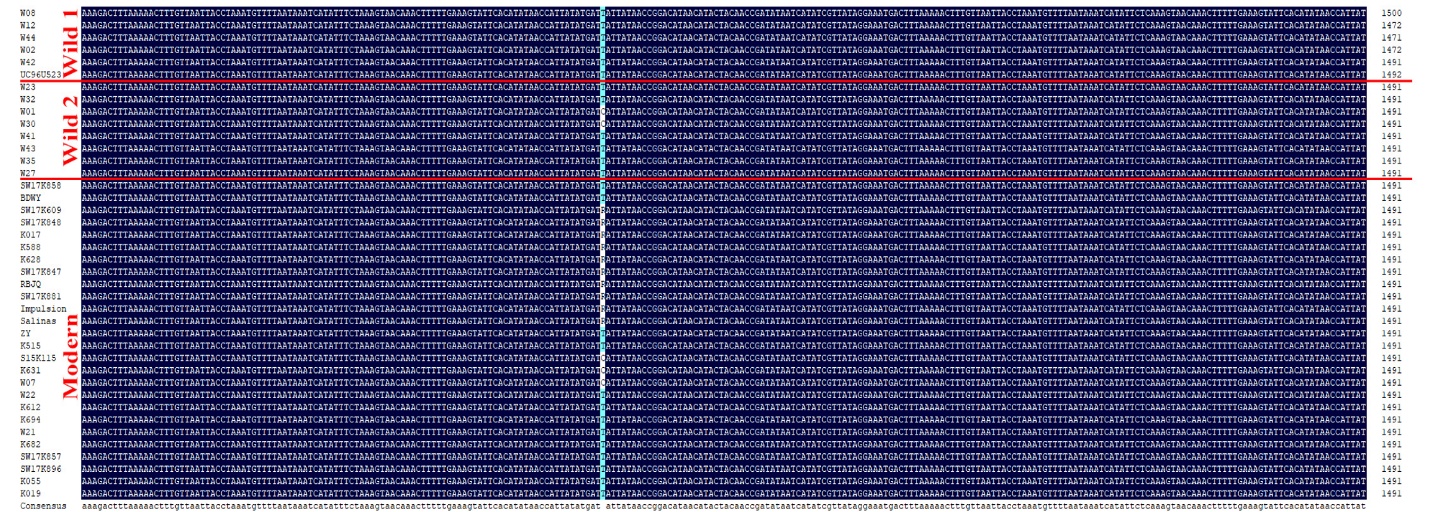

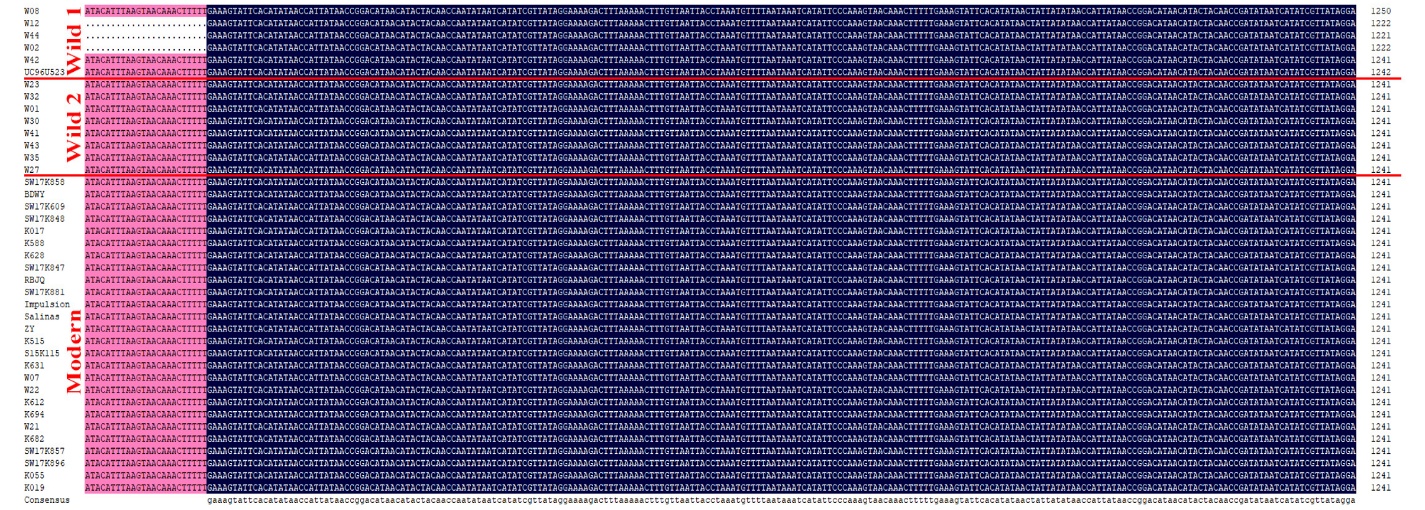


**
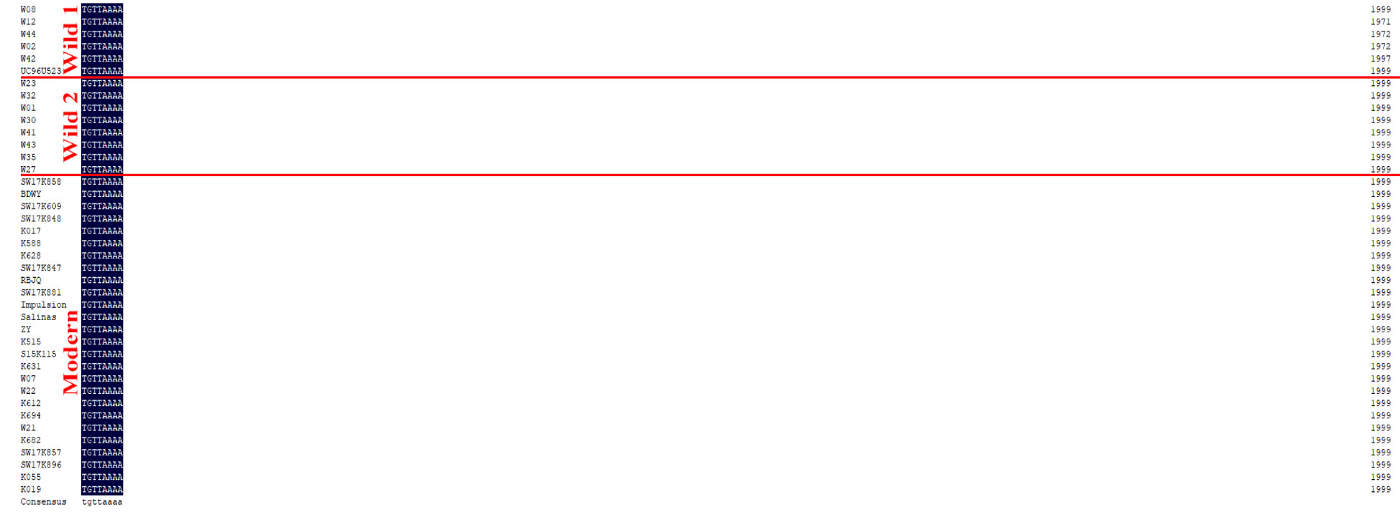
**

**Supplemental Figure S10** Promoter sequences analysis of *LsF3'H* between wild and modern lettuce groups.


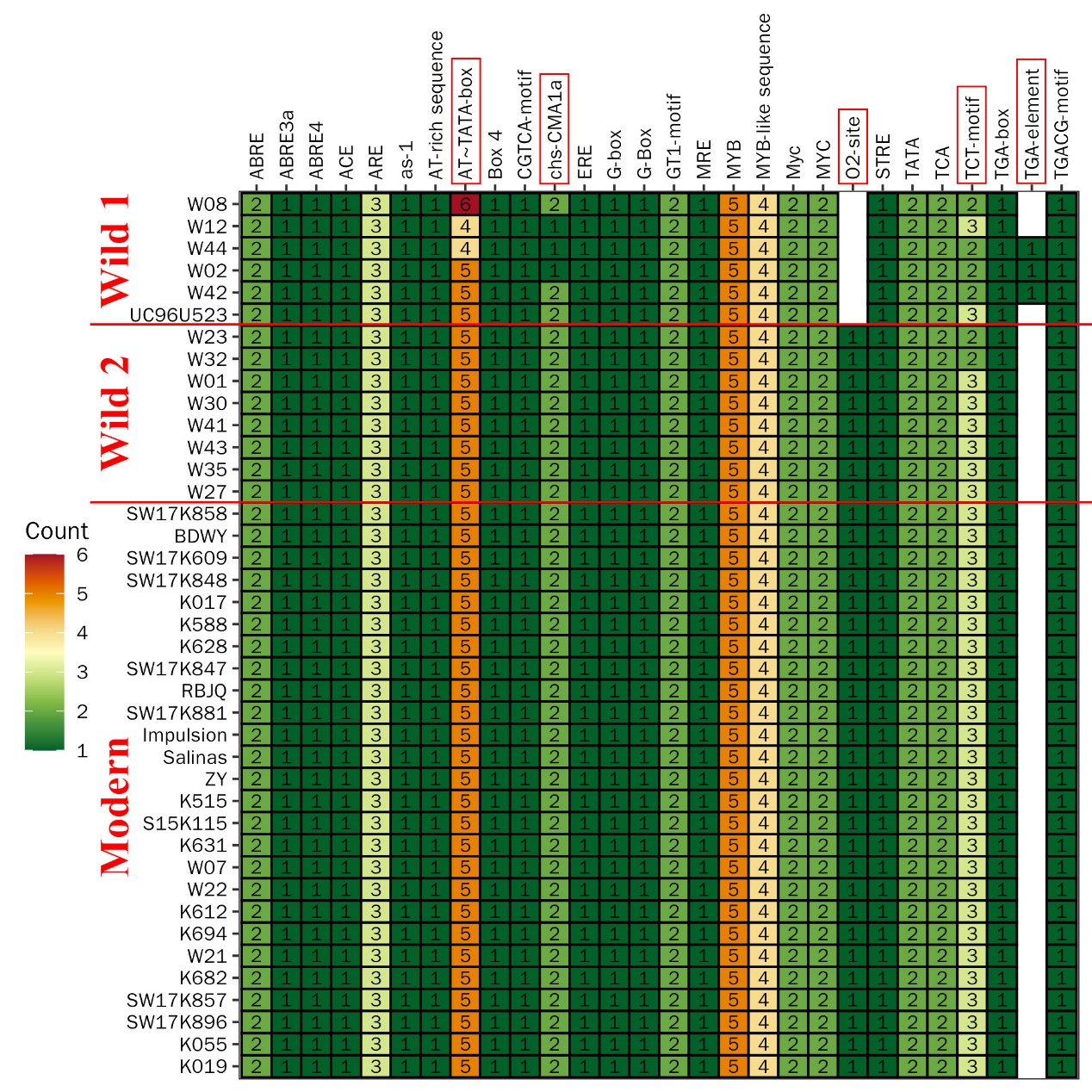


**Supplemental Figure S11** *Cis*-acting element analysis of LsF3'H promoter between wild and modern lettuce groups.

**
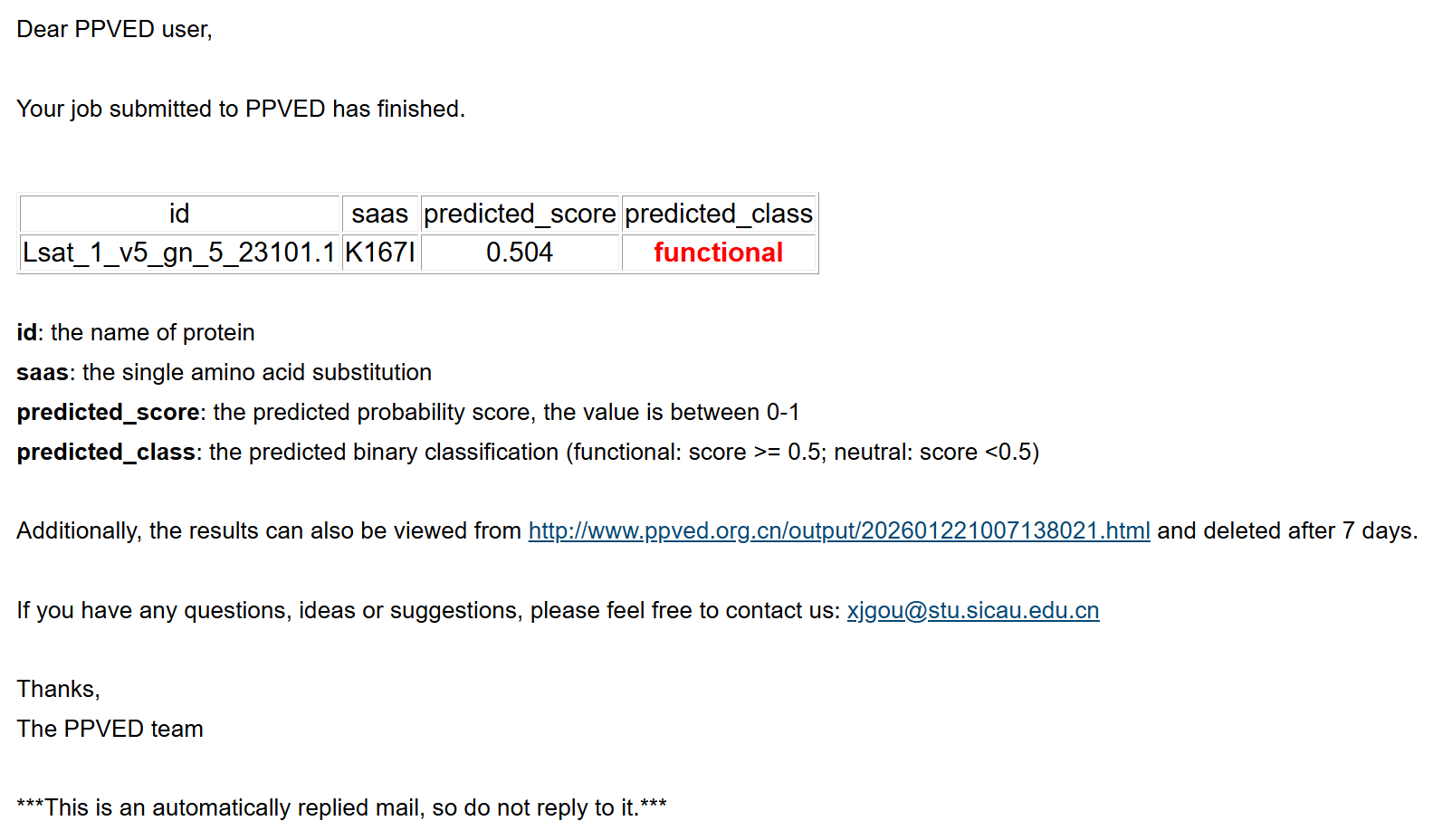
**

**Supplemental Figure S12** PPVED-based prediction indicates that the single non-synonymous mutation in LsF3'H has a potential functional impact.

**Supplemental Datasets Legends:**

**Supplemental Dataset S1** Genotypes and major morphological features of the lettuce accessions used in this study.

**Supplemental Dataset S2** Lettuce metabolites putatively identified by LC-MS.

**Supplemental Dataset S3** The VIP scores of metabolites in wild ancestors and modern lettuce cultivars.

**Supplemental Dataset S4** The volcano plot analysis of metabolites in wild ancestors and modern lettuce cultivars.

**Supplemental Dataset S5** The Mean Decrease Accuracy of metabolites in wild ancestors and modern lettuce cultivars.

**Supplemental Dataset S6** Re-sequencing information of wild ancestors and modern lettuce cultivars.

**Supplemental Dataset S7** Candidate genes detected by merging FST and CLR statistics.

**Supplemental Dataset S8** KEGG pathway enrichment of candidate genes selected by FST and CLR statistics

**Supplemental Dataset S9** F3'H gene expression levels in different growth stages and organs of the lettuce cultivar 'Ziguan'

**Supplemental Dataset S10** Cis-acting element analysis of *LsF3'H* promoter between wild and modern lettuce groups

**Supplemental Dataset S11** The variations in CDS region of *LsF3'H* in wild and modern lettuce groups
